# Bio‐Memristor Based on Clinical Bile Samples: A Proof‐of‐Concept Pilot Study for Detection of Gallbladder Cancer

**DOI:** 10.1002/advs.77893

**Published:** 2026-09-27

**Authors:** Junming Zhu, Yiyin Rong, Bai Sun, Ziwen Sun, Yifan Wang, Xiaobin Ren, Zelin Cao, Kaikai Gao, Jiawei Yu, Zhenkun Wang, Haoxuan Ma, Jichun Guo, Kai Cao, Mengrun Kang, Songling Wang, Fenggang Ren, Yi Lv, Qiang Lu

**Affiliations:** ^1^ Department of Abdominal Surgery The First Affiliated Hospital of Xi'an Jiaotong University Xi'an Shaanxi China; ^2^ Frontier Institute of Science and Technology, and Interdisciplinary Research Center of Frontier Science and Technology Xi'an Jiaotong University Xi'an Shaanxi China; ^3^ Micro‐and Nano‐technology Research Center State Key Laboratory for Manufacturing Systems Engineering Xi'an Jiaotong University Xi'an Shaanxi China; ^4^ State Key Laboratory of Structural Chemistry Fujian Institute of Research on the Structure of Matter Chinese Academy of Sciences Fuzhou China; ^5^ National Local Joint Engineering Research Center for Precision Surgery and Regenerative Medicine The First Affiliated Hospital of Xi'an Jiaotong University Xi'an Shaanxi China

**Keywords:** bio‐electronic interface, intelligent medicine, medical detection, memristive signature, memristor

## Abstract

Gallbladder cancer (GBC) is the most common malignant tumor of the biliary system, and the 5‐year survival rate of GBC patients is only 5% due to delayed diagnosis. The sensitivity and specificity of existing clinical testing equipment for gallbladder malignancy remain insufficient. In this proof‐of‐concept pilot study, we developed a bio‐memristor featuring an Ag/bile/fluorine‐doped tin oxide structure using clinically sourced bile samples from a small clinical cohort and characterized its resistive switching behavior. The memristive electrical signatures, particularly negative peak current and the high‐resistance‐state to low‐resistance‐state ratio, differed systematically across people with normal gallbladder, cholelithiasis patients, and GBC patients, enabling a two‐step detection strategy. Within this pilot cohort, the detection performance was encouraging. These findings establish the technical feasibility of memristor‐based bile detection for gallbladder cancer and provide a foundation for developing detection technology that is expected to complement existing clinical approaches to improve gallbladder cancer identification.

## Graphical abstract

In this work, we present a bile‐based memristor hardware framework that enables direct, label‐free identification of cholelithiasis and gallbladder cancer at the device level. The Ag/bile/FTO memristor features stable resistive switching and disease‐specific electrical responses, providing a conceptual foundation for circuits and an integrated ERCP‐compatible detection system.

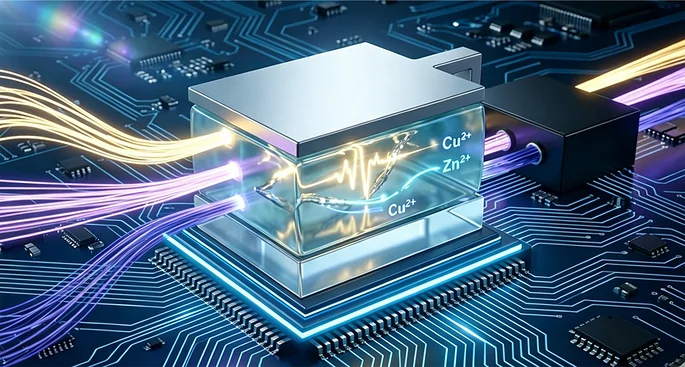

## 1 Introduction

Gallbladder cancer (GBC) is the most prevalent and highly malignant subtype of biliary tract cancer [1, 2, 3, 4]. Although the overall 5‐year survival rate for patients with early‐stage GBC can reach 62% to 75%, the corresponding rate for the entire patient population is merely 5% [5]. The primary reason lies in the insidious nature of its early symptoms, which often leads to late‐stage diagnosis, by which time patients have already missed the optimal window for radical surgical intervention. In addition, GBC has poor sensitivity to radiotherapy and chemotherapy. Once the opportunity of radical surgery is delayed, the prognosis of patients is very poor [6, 7, 8].

From the perspective of Pathology, GBC is often secondary to malignant transformation of benign biliary diseases, and cholelithiasis is recognized as the primary risk factor. Long‐term cholelithiasis not only produces mechanical irritation to gallbladder mucosa, but also cause chronic cholecystitis. This can lead to repeated injury and repair of gallbladder epithelial cells, promote abnormal cell proliferation and gene mutation accumulation, and eventually lead to cancer [9, 10]. In this inflammatory environment, gallbladder mucosa will undergo thickening, edema, fibrosis, and even gland hyperplasia, which will cover the flat or adherent invasive growth of early GBC [1, 5, 11].

This pathological change and its accompanying morphological masking effect pose fundamental challenges for current testing equipment. At the serological level, conventional tumor markers such as CA19‑9 lack sufficient sensitivity and specificity for the diagnosis of GBC [12]. Radiologically, ultrasound exhibits insufficient sensitivity in distinguishing minute gallbladder tumors (≤1 cm) from non‑protuberant thickening of the gallbladder wall [1, 11]. Magnetic Resonance Cholangiopancreatography often shows highly overlapping signal characteristics between benign and malignant biliary lesions on T1WI, T2WI, and DWI sequences, limiting its ability to accurately differentiate them [13]. Although Endoscopic Retrograde Cholangiopancreatography (ERCP) offers both diagnostic and therapeutic benefits, its efficacy in detecting minute lesions is constrained by the insufficient sensitivity of gross visual inspection and conventional biopsy methods [14]. Consequently, the development of rapid and accurate detection technologies to complement existing methods remains an unmet clinical need.

However, this very pathological process also provides a unique opportunity for identification. The progression from chronic inflammation to cancer is accompanied by the direct release of tumor‐derived cells, cell‐free nucleic acids, proteins, and altered metabolites into the bile [5, 15, 16]. Bile, being in direct contact with the biliary epithelium, continuously accumulates these molecular alterations from the entire pancreaticobiliary system, including areas difficult to reach by ERCP [17, 18]. Therefore, bile represents a unique and information‐rich detection medium that can reflect pathological changes earlier and more directly than serum markers, enabling more responsive identification of minor lesions. This transforms the pathological challenge into a detection opportunity, and calls for a sensing technology with sufficient sensitivity to read out these molecular signatures.

To meet this need, as the fourth basic electronic component, the memristor stands out among nonlinear resistance devices with its charge storage capacity and unique resistance memory characteristics [19, 20, 21, 22]. Memristors can convert specific micro biochemical signals into quantifiable and high‐resolution electrical responses, showing great potential in the fields of biological detection and health monitoring [23]. Currently, the memristor‐based sensing platform has undergone validation in multiple medical detection scenarios, fully demonstrating its potential for clinical translation [24, 25, 26, 27]. The Ag/c‐YY NW/Ag memristor achieves integrated sensing and pulse coding through humidity‐controlled proton‐coupled ion migration, enabling non‐invasive differentiation of normal, asthmatic, and restrictive breathing patterns [25]. The TaO_x_/Ta_2_O_5_ memristor eliminates the need for complex threshold‐judgment circuits via threshold‐switching and non‐volatility, enabling flexible and tunable bedside diagnostic pH detection [26]. The Ag/Ta_2_O_5_/ITO threshold switch type memristor and the integrated cell memristor architecture of Zn‐Ag primary battery enable self‐powered ion concentration sensing and frequency coding spike output, which can be used in medicine for real‐time monitoring of electrolyte changes in body fluids [27]. The above research indicates that memristors can extract high‐resolution information from various biological inputs, making them highly suitable for detecting subtle molecular changes in bile during gallbladder carcinogenesis.

In this study, we developed an Ag/bile/ Fluorine‐doped tin oxide (FTO) structured biological memristor and characterized its resistive switching behavior. By leveraging the differential electrical responses of bile under various pathological conditions, we established a correlation between the pathological features of bile and the resistive switching behavior of the memristor, enabling differentiation between gallbladder cancer and non‐malignant biliary conditions. This approach offers rapid response and high integration potential. A conceptual design for integration with ERCP equipment was also developed to illustrate a potential future application pathway. These findings provide a foundation for further investigation of memristor‐based bile detection for gallbladder cancer.

## 2 Results and Discussion

### 2.1 Device Structure and Chemical Composition

**FIGURE 1:**
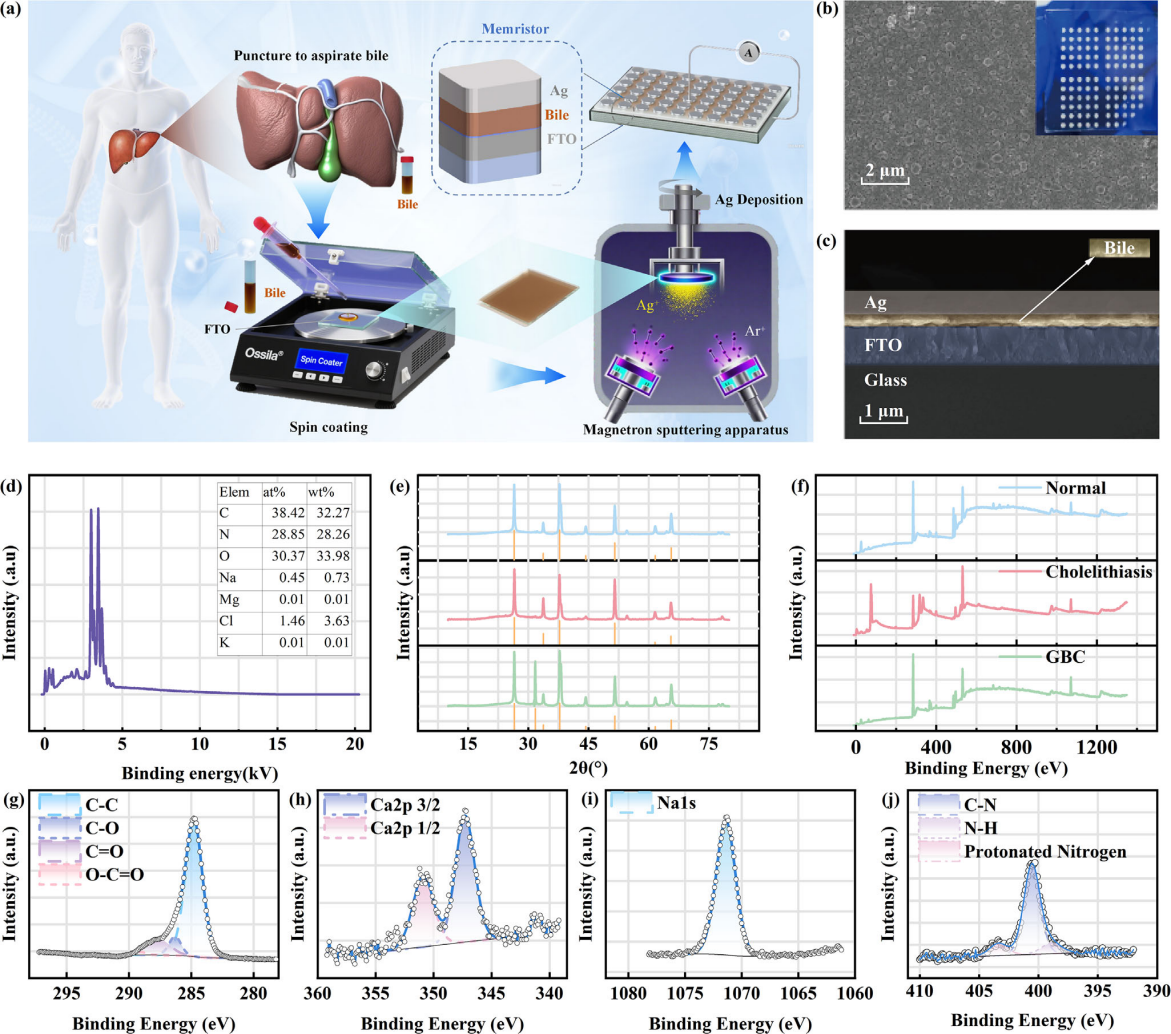
Fabrication and Material Characterization of the Ag/bile/FTO Memristor. (a) Schematic illustration of the device fabrication process. (b) Planar SEM image of the bile thin film, with an inset optical photograph of the device array. (c) Cross–sectional SEM image of the memristor, revealing a distinct Ag/Bile/FTO sandwich structure. (d) EDS spectrum and quantitative elemental composition analysis of the control bile thin film. (e) Comparative XRD patterns of bile thin films derived from people with normal gallbladder (control), cholelithiasis patients, and GBC patients. (f) Comparative full XPS spectra of the three types of bile thin films. (g–j) High–resolution XPS spectra of the control bile thin film, corresponding to the C1s, Ca2p, Na1s, and N1s orbitals, respectively.

In this study, we fabricated a typical sandwich‐structured Ag/bile/FTO memristor. Initially, bile samples were collected from people with normal gallbladders, patients with cholelithiasis, and those with GBC. After spin–coating, silver electrodes were deposited on the bile film through magnetron sputtering, completing the device fabrication (Figure 1a). To reveal the microstructure and composition of the functional layer in Ag/bile/FTO memristors, we performed multi–scale characterization.

The planar SEM image (Figure 1b) reveals a continuous and structurally homogeneous surface of the bile film, with no evident pores or agglomeration, indicating excellent film‐forming quality. The inset of Figure 1b shows a macro photograph of the memristor array structure. The cross‐sectional SEM image (Figure 1c) reveals a typical sandwich structure, with a bile film thickness of approximately 200 nm and a tightly bound interface. To examine the uniformity of composition, energy dispersive spectroscopy (EDS) was conducted on both cross–sectional and plan views. Figure S1a–l demonstrates that the distribution of C, N, O, and Ag is spatially consistent with the designed device architecture. Figure 1d shows the EDS spectrum of the control bile layer. Control bile is primarily composed of C, N, and O, with contents of 32.27%, 28.26%, and 33.98%, respectively, forming the organic backbone of bile acids and phospholipids. Meanwhile, trace amounts of Cl, Na, and K are consistent with the physiological electrolyte characteristics of bile.

To guarantee reliable resistive switching performance and stable detection capacity of the fabricated memristors, we systematically evaluated the intra‐batch and inter‐batch reproducibility of our device fabrication protocol. Physical morphology and statistical characterizations were performed on Ag/bile/FTO memristors from five independent production batches (Batch 1–5), as summarized in Figure S2. Cross‐sectional SEM images in Figure S2a verify the formation of compact, well‐defined layered architectures across all batches. Thickness statistics collected from 30 individual devices (6 devices per batch) reveal highly uniform bile functional layers with a global mean thickness µ of 202.23 nm and an ultra‐low coefficient of variation (CV) of only 0.031, demonstrating outstanding inter‐batch thickness homogeneity (Figure S2b–f,l). Meanwhile, grayscale roughness analysis of planar SEM micrographs via ImageJ (Figure S2g–k) is cross‐validated by direct topography and roughness measurements from AFM characterization (Figure S2p–u). The surface roughness Ra of all batches fluctuates within a narrow range of 13.7–22.2 nm, further confirming that this fabrication strategy preserves exceptional surface flatness and morphological consistency even during large‐scale device fabrication (Figure S2o). Such excellent structural reproducibility lays a robust material foundation for constructing memristor platforms with highly uniform electrical response profiles in subsequent detection.

The structural phase of the active layer was further examined by X–ray diffraction (XRD) (Figure 1e). XRD testing shows that the three types of bile‐based devices share common diffraction peaks at 37.80°, 26.50°, 31.72°, and 51.56°, corresponding to the underlying FTO substrate [28, 29, 30]. In contrast, the bile functional layer exhibits broad diffuse bulges near 30°, stemming from the amorphous and weakly crystalline arrangement of bile acids, phospholipids, and other components, predominantly in an amorphous state. The bile membrane of GBC shows a sharp characteristic peak at 31.72°. Its components form a more ordered crystalline structure, which is related to the precipitation of organic crystal forms and the aggregation of trace salts.

We further investigated the chemical states by X‐ray photoelectron spectroscopy (XPS). The XPS survey spectrum reveals significant differences in the chemical state of the bile functional layer under various pathological conditions (Figure 1f). We further investigated the chemical states by high‐resolution X‐ray photoelectron spectroscopy (XPS). In control bile, C mainly exists in the form of C─C bond at 284.8 eV, with a weak C═O signal (Figure 1g). The binding energies of Ca^2^^+^ ions and Na^+^ ions are approximately 347 and 1071 eV, respectively (Figure 1h,i). N1s can be fitted to C─N, N─H, and protonated nitrogen signals (Figure 1j). The XPS results of the bile device from cholelithiasis shown in Figure S3 indicate an increase in the proportion of C**═**O bonds, accompanied by the appearance of characteristic double peaks at 347.3 and 350.9 eV for Ca 2p. In Figure S4, the peak intensities of Na and Cl in the bile device from GBC are significantly increased, and the C─O bond shifts to 286.0 eV with enhanced signal. These results confirm that the memristor is a chemically integrated hybrid layer and demonstrate significant differences in pathological characteristics at the molecular electronic structure level.

The multi–scale characterization results of the Ag/bile/FTO memristor functional layer indicate that the device preparation is consistent with expectations, providing solid support for its reliable electrical performance. Meanwhile, characterization techniques such as XPS and XRD reveal the compositional and structural differences of bile under various pathological conditions. This lays the foundation for the application of this memristor in disease identification and detection.

### 2.2 Resistive Switching Behavior and Device Stability

**FIGURE 2:**
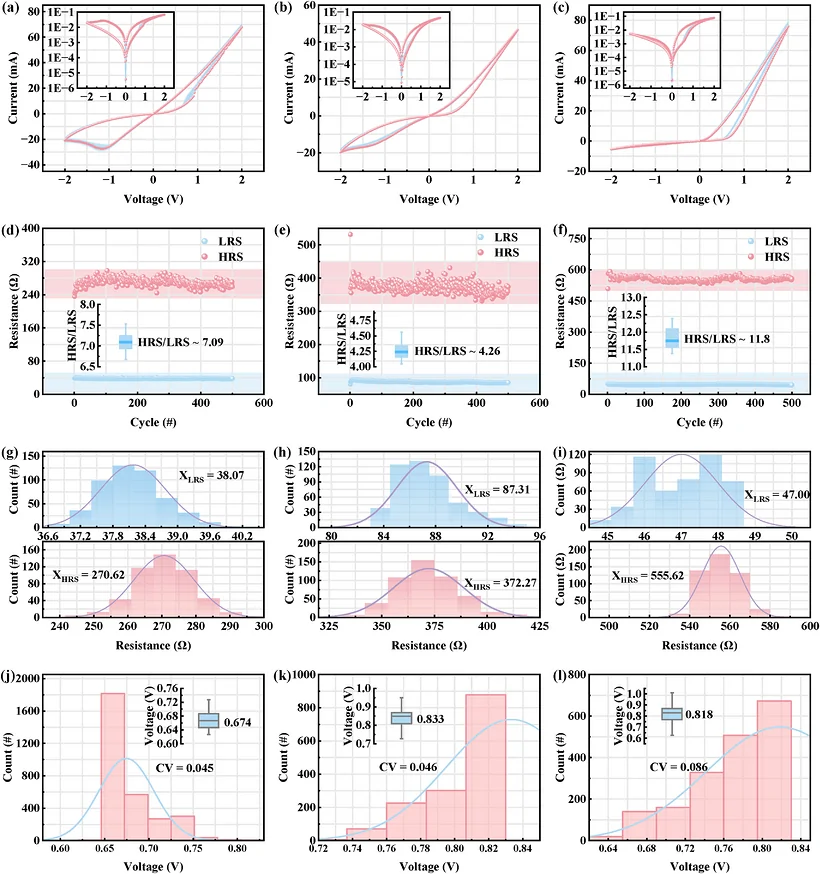
Resistive switching characteristics and stability of bile–based biomemristors. (a–c) Representative I–V curves under cyclic voltage sweeps (0 V → 2.0 V → −2.0 V → 0 V) for devices with (a) control bile, (b) cholelithiasis bile, and (c) GBC bile. Insets show semi–logarithmic plots of the corresponding *I–V* curves, highlighting the bipolar resistive switching behavior. (d–f) Cycle–to–cycle endurance over 500 switching cycles, with high resistance state (HRS, red) and low resistance state (LRS, cyan) distributions, and corresponding on/off ratios. (g–i) Statistical distributions of LRS (cyan) and HRS (red) extracted from the endurance tests, showing near–Gaussian distributions with narrow dispersion. (j–l) Distributions of SET voltages (transition from HRS to LRS) for the three device groups, with coefficient of variation (CV) values indicating low device–to–device variability and good switching uniformity.

The study involves the preparation of bile samples collected clinically into Ag/Bile/FTO structured biomemristors and the systematic evaluation of the resistive switching behavior of devices with different pathological bile. Figure 2a–c illustrates the *I–V* curves of three types of devices under a cyclic voltage of 0 V → 2.0 V → –2.0 V → 0 V. All devices exhibited typical bipolar resistive switching characteristics and could withstand 500 switching cycles stably, demonstrating excellent cycling stability. All three sets of devices exhibited clear resistive switching hysteresis loops, but the current response varied significantly depending on the pathological state. The *I–V* curve of the control bile device was well–symmetric, and the current response was stable. The current amplitude of the cholelithiasis device was significantly reduced. The set–point transition of the GBC device was steeper, and the negative current further decreased, indicating that changes in pathological bile composition had a significant impact on the electric field–resistance state response.

Figure 2d–f depicts the resistance evolution curves over 500 cycles. The control bile device exhibits a high resistance state (HRS) of approximately 270 Ω, a low resistance state (LRS) of approximately 38 Ω, and a switching ratio of approximately 7.09. The cholelithiasis device shows an HRS of approximately 372 Ω, an LRS of approximately 87 Ω, and a switching ratio of approximately 4.26. The GBC device has an HRS of approximately 555 Ω, an LRS of approximately 47 Ω, and a switching ratio as high as 11.8, which is the highest among the three. The resistance ratio and distribution of the three groups of devices are highly correlated with the pathological state of bile, confirming that the devices exhibit specific electrical responses to different pathological bile conditions. This allows for effective differentiation between physiological and pathological states, indicating potential for clinical detection [26, 31].

To verify the resistive switching stability of the device, we statistically analyzed the resistance state distribution of three types of bile memristors (Figure 2g–i). All resistance states approximately followed a Gaussian distribution. The low resistance state (LRS) and high resistance state (HRS) of control bile devices were centered at 38.07 and 270.62 Ω, respectively, with a narrow and symmetrical distribution. The center values of the cholelithiasis devices were 87.31 and 372.27 Ω, with an overall shift toward higher resistance and increased dispersion in the HRS. The LRS of the GBC device was 47.00 Ω, and the HRS reached as high as 555.62 Ω, with a widening distribution within a limited range. The resistance state transitions of devices with different pathologies exhibited good controllability and repeatability, which can reduce parameter fluctuations between cycles and provide guarantees for signal differentiation.

The uniformity of the distribution of the set voltage (SET) is a core indicator of the consistency of memristors [19, 32]. This study selected 30 sets of clinical samples to complete statistical analysis of SET voltage, using the coefficient of variation (CV) to characterize dispersion. The results showed that the CV values for the control group and the cholelithiasis group were as low as 0.045 and 0.046, respectively, demonstrating excellent stability. The CV value for the GBC group was approximately 0.086, which was slightly elevated but still within a controllable range. All three types of devices exhibited low random fluctuations in SET voltage, reducing parameter differences between samples and enhancing the batch stability and versatility of the system. This provides a crucial electrical performance foundation for precise classification of gallbladder diseases. Figures S6–S8 display representative multi–device *I–V* curves, indicating good inter–device reproducibility during the fabrication process. Overall, the stable and narrowly distributed SET voltages as well as the well–reproducible *I–V* curves of multiple devices jointly demonstrate excellent device–to–device uniformity of the bile memristors [33]. Overall, the excellent cycling stability, good consistency among devices, and distinct electrical response differences among control, cholelithiasis, and GBC bile devices support consistent discrimination between these three sample groups within this pilot cohort. This provides a technical basis for further investigation of memristor‐based bile detection for gallbladder disease.

### 2.3 Key Indicator Verification and Detection Performance

To construct a memristive sensing system tailored for precise classification of gallbladder diseases, we further screened and evaluated the key features with significant differentiation in the electrical response of the device. Based on 30 sets of clinical bile samples (including three categories: control, cholelithiasis, and GBC), memristors were prepared. Multidimensional electrical characteristics were systematically extracted to establish a feature library and carry out classification performance evaluation.

**FIGURE 3:**
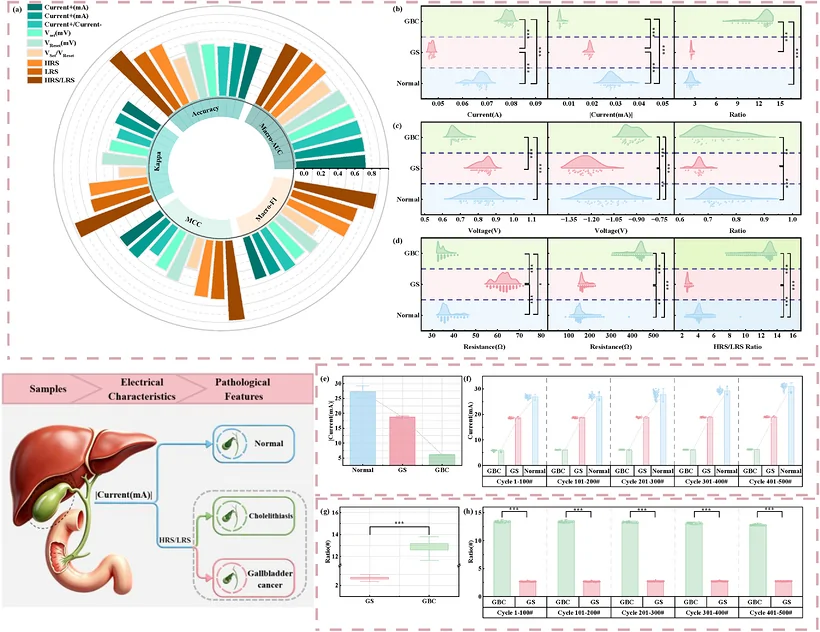
Screening of memristive electrical signatures and construction of a two‐step detection system for gallbladder diseases. (a) Radar chart of multi‐index classification performance evaluating the discriminative ability of different electrical features. (b–d) Statistical distributions and intergroup comparisons of key features related to current, voltage, and resistance across three sample groups. (e–h) Validation of the two‐step detection system using dual biomarkers (schematic shown on the left): first, negative peak current distinguishes control from pathological samples. then, the HRS/LRS ratio differentiates GBC from cholelithiasis. (e) Quantitative comparison of negative peak current among the three groups. (f) Cycle‐to‐cycle stability of negative peak current over 500 switching cycles, maintaining consistent intergroup gradients. (g) Statistical comparison of HRS/LRS ratios between cholelithiasis and GBC groups. (h) Cycle‐to‐cycle stability of the HRS/LRS ratio over 500 cycles, with highly significant differences between the two pathological groups throughout all intervals. Statistical significance is determined by Kruskal‐Wallis test followed by Dunn's multiple comparisons test. *p* < 0.001, *p* < 0.01, *p* < 0.05.

The radar chart analysis of multi‐index classification performance (Figure 3a) reveals that different electrical features exhibit differentiated contributions in disease differentiation tasks. Among them, the HRS/LRS switch ratio, negative peak current, and HRS stand out in key evaluation metrics such as accuracy, Kappa coefficient, Matthews correlation coefficient (MCC), Macro‐F1, and Macro‐AUC, demonstrating high potential for distinguishing among the three types of samples. To quantify the discriminative power of distinct electrical signatures for disease stratification, we first performed statistical effect size evaluation on all samples. As illustrated in Figure S9a,b, at both the device‐level and patient‐level dimensions, signatures including the HRS/LRS switching ratio and negative peak current yield an extremely large Cohen's f effect size exceeding 2.7, accompanied by statistical power above 0.9999. These values substantially outperform those of other voltage and current parameters. These statistical results fundamentally validate the outstanding reliability of these electrical metrics as core classification features. Based on the aforementioned performance evaluation, we assessed the inter‐group differences in current, voltage, and resistance dimensions among bile samples with different pathological states. We employed the Kruskal‐Wallis test for non‐parametric statistical analysis of the differences in electrical characteristics among the three groups of samples. The results indicated that all the electrical characteristics included in the analysis exhibited highly significant statistical differences (p <0.001) among the three groups of samples.

Figure 3b–d illustrates the distribution of key electrical characteristics and inter‐group differences for three types of bile samples. In terms of current response, the GBC group exhibited the highest positive peak current, with a distribution concentrated between 0.07 and 0.09 A, significantly higher than that of the control group and cholelithiasis group. This suggests that bile from GBC can promote the formation of Ag conductive filaments under positive bias. The absolute value of negative peak current decreases with the severity of the lesion, with the highest in the control group, followed by the cholelithiasis group, and the lowest in the GBC group, which is related to the differences in bile endogenous substance composition. The positive‐to‐negative peak current ratio in the control group and cholelithiasis group exhibited similar, albeit slightly lower, values, whereas it was significantly elevated in the GBC group, forming a highly significant difference from the two benign groups and possessing the potential to differentiate malignant lesions. Voltage‐related characteristics exhibit pathological dependence with benign convergence and malignant differentiation. The distribution of SET voltage, RESET voltage (transition from LRS to HRS), and voltage ratio in the control group and cholelithiasis group overlaps highly with no statistical difference. In the GBC group, the SET voltage shifts toward lower voltage ranges, while the RESET voltage shifts toward 0 potential direction. The distribution of voltage ratio widens and shifts toward lower values, with significantly enhanced bidirectional bistable dynamics asymmetry, echoing the bidirectional differentiation pattern of current characteristics. Among the resistance‐related characteristics, the LRS resistance in the cholelithiasis group was significantly elevated compared to both the control group and the GBC group, showing statistical differences. The HRS resistance and switch ratio exhibited a benign convergence toward malignant differentiation, with the distribution of HRS resistance in the control group and the cholelithiasis group highly overlapping, and the switch ratio concentrated in the 2–4 range. The HRS resistance in the GBC group was significantly elevated, with the distribution broadening to 300–500 Ω, and the switch ratio increasing to the 10–16 range, forming a highly significant difference from the previous two groups.

Figure 3a shows that the negative peak current and HRS/LRS switching ratio excel in key classification indicators, and Figure 3b–d confirm significant differences and good stability between different pathological samples, thus they are selected as core detection indicators. To intuitively visualize the separation boundaries of the two core features, we further analyzed feature distribution spaces and confusion matrices. As shown in Figure S9c, the two‐dimensional scatter plot built from negative peak current and HRS/LRS ratio achieves distinct clustering separation of control, cholelithiasis and GBC samples via two cut‐off thresholds (|Current| = 0.0224 A and HRS/LRS Ratio = 2.9232). The corresponding three‐class confusion matrix (Figure S9d) reveals exceptional detection accuracy at the patient level, with all 10 GBC samples correctly identified without any missed or misidentification. Based on negative peak current and HRS/LRS ratio, a two‐step detection system for gallbladder diseases has been established (schematic diagram on the left). This system relies on the differential memristive electrical responses induced by bile with different pathologies to achieve stratified identification of three types of samples. The first step utilizes negative on‐state current to distinguish between control and pathological samples. The negative on‐state current of control bile‐based devices is significantly higher than that of the cholelithiasis and GBC groups, forming a clear boundary between control and pathological signals. The second step employs the HRS/LRS switching ratio to differentiate between GBC and cholelithiasis samples.

To validate the discriminative power and stability of key electrical features, we analyzed the negative peak current and HRS/LRS ratio across bile devices with different pathologies. As shown in Figure 3e,g, these two features exhibit distinct pathological differences: the negative peak current decreases in the order control > cholelithiasis > GBC, while the ratio is significantly higher in GBC devices than in cholelithiasis ones. Further cyclic analysis over 500 switching cycles, with every 100 cycles segmented (Figure 3f,h), reveals that the negative current maintains a consistent gradient among the three groups throughout all intervals, and the ratio difference between the two pathological groups remains highly significant at each stage. These results demonstrate that both features possess excellent discriminative ability and cycle‐to‐cycle stability, providing robust electrical signatures for reliable gallbladder disease discrimination. We calculated the statistical power at each analytical stage. As shown in Figure S9e, the power curve of each procedure quickly crosses the conventional significance threshold of 80% with increasing Cohen's d to verify the statistical reliability of the above classification model based on limited clinical samples. Figure S9f further demonstrates that the experimentally observed effect size (Observed d) is far larger than the theoretical d required to achieve 80% statistical power, with the maximum ratio (d_obs / d_80%) close to 12. From the perspective of statistical power analysis, this suggests that the observed separation is unlikely to result from random variation in this small sample set, although independent external validation is still required.

Building on the two‐step detection workflow established above, we evaluated the overall classification performance using the patient as the analytical unit. The 90 measured devices corresponded to technical replicates from 30 patients (three per patient) and were therefore not independent clinical samples. To avoid spurious inflation of the effective sample size, we aggregated the three technical replicates into a single patient‐level mean for all primary analyses, after confirming their acceptable repeatability (Figure S10a–c). To rigorously eliminate information leakage, we implemented a Leave‐One‐Out Cross‐Validation (LOOCV) strategy (30 folds). In each fold, all devices of the held‐out patient were strictly excluded from the training set, and the two cutoff values were re‐optimized within the training fold by maximizing Youden's J index, before being applied to the held‐out patient to obtain unbiased predictions (Figure S10).

At the patient level, Step 1 (separating control from pathological samples) yielded a point‐estimate sensitivity of 100% (20/20) and a specificity of 90% (9/10), correctly classifying 29 out of 30 patients (Figure S10e). Step 2 (distinguishing cholelithiasis from GBC) yielded a sensitivity of 100% (10/10) and a specificity of 90% (9/10), with one cholelithiasis patient misclassified as GBC (Figure S10g). The aggregated three‐class confusion matrix demonstrated an overall accuracy of 93.3% (28/30), with the two misclassifications confined to patients with boundary‐level electrical characteristics (Figure S10h).

The cutoff values exhibited remarkable stability across iterations, with a Step 1 cutoff (current) of 0.0224 ± 0.0028 A and a Step 2 cutoff (HRS/LRS) of 2.9232 ± 0.0675 (Figure S10d, f). This narrow spread reinforces the robust threshold behavior and intrinsic group‐level separation of our electrical readouts. The patient‐level ROC analysis yielded an AUC of 1.000 for both steps (Figure S10j,k), and in 10,000 patient‐level bootstrap resamples, both AUC values remained at 1.000 in every iteration.

Stratified bootstrap resampling further provided point estimates and 95% confidence intervals (CIs) visualized in the forest plot (Figure S10i). It is noteworthy that the 95% CIs lower bounds of specificity and PPV exhibited significant downward extension, which is statistically attributed to the small effective sample size under LOOCV and the inherent clinical imbalance of pathological subtypes (e.g., rare GBC cases). This does not imply model failure; rather, the high point estimates with upper CIs approaching 1.0 robustly confirm the model's clinical feasibility, while explicitly charting the need for larger, multi‐center external cohorts to narrow these intervals. Furthermore, a supplementary analysis using the median of the three technical replicates correctly classified 29 out of 30 patients. These patient‐level analyses demonstrate the excellent internal consistency and promising discriminatory capacity of the memristor‐enabled detection strategy in this pilot cohort. Confirmation of generalizability requires independent external validation in larger cohorts.

Even with this broadening, performance metrics remained consistent across repeated measurements within this dataset. Collectively, these multi‐dimensional analyses demonstrate the internal consistency and promising discriminatory capacity of the memristor‐enabled detection strategy within this pilot cohort (N = 30). Confirmation of generalizability requires independent external validation in larger, multi‐center cohorts.

### 2.4 Assessment of Covariates and Potential Confounders

Because the three groups differed in demographic and clinical characteristics (Table S26), we examined whether these covariates could account for the observed intergroup differences in the two core signatures. In the full cohort (N = 30), the negative peak current (I^−^) correlated significantly only with WBC count (Spearman *ρ* = −0.61, *P* < 0.001), whereas TBil, DBil, ALT, AST, and CRP all showed null associations (|*ρ*| ≤ 0.24, *P* ≥ 0.20) (Table S7). By contrast, the HRS/LRS ratio correlated significantly with age (*ρ* = +0.57, *P* < 0.001), TBil (*ρ* = +0.50, *P* = 0.005), DBil (*ρ* = +0.65, *P* < 0.001), and AST (*ρ* = +0.39, *P* = 0.031) (Table S7). None of these associations persisted within groups (all within‐group *P* > 0.30, Tables S8 and S9). The group means moved in parallel across groups, with the HRS/LRS ratio falling from 4.18 in the control group to 2.69 in cholelithiasis and rising to 12.48 in GBC, and TBil, DBil, and AST following the same pattern (Table S10). This pattern is characteristic of an ecological correlation, in which parallel group means appear correlated even when the underlying individuals do not covary. Consistent with this interpretation, TBil spanned a 17‐fold range (13.2–229.0 µmol/L) within the GBC group, yet neither bilirubin nor any liver enzyme correlated with either signature.

Gender differed markedly across groups (90%, 50%, and 10% male in the control, cholelithiasis, and GBC groups, respectively, Fisher exact *P* = 0.001, Table S11), reflecting the established female predominance of gallbladder cancer, and is therefore entangled with the disease state itself. Gender nonetheless showed no measurable effect on either signature where a within‐group test was possible. In the cholelithiasis group, the only group with balanced gender representation, the negative peak current was 0.0187 ± 0.0010 A in men and 0.0190 ± 0.0004 A in women (Mann–Whitney U, *P* = 0.69), and the HRS/LRS ratio was 2.75 ± 0.16 versus 2.63 ± 0.12 (*P* = 0.31) (Table S12). In the partial correlation analysis (Table S13), age was still significantly correlated with HRS/LRS ratio after controlling for gender (*ρ* = +0.53, P = 0.003), but not significantly correlated with negative peak current (*ρ* = −0.27, *P* = 0.16). However, gender was not correlated with either of the two characteristics after controlling for age (*ρ* = −0.11, *P* = 0.56, and *ρ* = +0.10, *P* = 0.58). As with the clinical variables, these gender associations mirrored the group gradient rather than individual‐level gender effects. To test this directly, we repeated the between‐group comparisons underlying the two detection steps within a single gender (Table S14). Among men, the Step 1 separation between the control and cholelithiasis groups persisted, with the negative peak current at 0.0275 ± 0.0031 versus 0.0187 ± 0.0010 A (*P* = 0.001, Cohen's d = −3.36) and the HRS/LRS ratio at 4.18 ± 0.39 versus 2.75 ± 0.16 (*P* = 0.001, *d* = −4.33). Among women, the Step 2 separation between the cholelithiasis and GBC groups persisted, with the negative peak current at 0.0190 ± 0.0004 versus 0.0064 ± 0.0008 A (*P* = 0.001, *d* = −19.16) and the HRS/LRS ratio at 2.63 ± 0.12 versus 12.41 ± 1.20 (*P* = 0.001, *d* = +9.91), and the bootstrap‐averaged cut‐off values (0.0224 A and 2.9232) reproduced the overall leave‐one‐out performance within both gender strata, with 29 of 30 patients correctly classified at Step 1 and 19 of 20 at Step 2. The only misclassified patients were the boundary cases already identified by the leave‐one‐out analysis, one control patient at Step 1 and one cholelithiasis patient at Step 2, both male.

Age was also treated as a categorical variable to further exclude its influence. Because 60 years corresponds to both the cohort median age (59 years) and the conventional threshold used to define older patients, the cohort was dichotomized into younger (<60 years, *n* = 17) and older (≥60 years, *n* = 13) strata. Within each disease group, neither signature differed between the two age categories (Table S15). In the control group, the negative peak current was 0.0267 ± 0.0038 A in younger and 0.0283 ± 0.0017 A in older patients (*P* = 0.69), and the HRS/LRS ratio was 4.20 ± 0.49 versus 4.15 ± 0.24 (*P* = 1.00). In the GBC group, the corresponding values were 0.0061 ± 0.0002 versus 0.0065 ± 0.0008 A (*P* = 0.83) and 12.85 ± 0.40 versus 12.32 ± 1.36 (*P* = 1.00). In the cholelithiasis group, the single older patient (ratio 2.71) fell within the range of the nine younger patients (2.49–2.97). A naive full‐cohort comparison of age categories likewise mirrored the group gradient rather than an age effect. The overall difference in the HRS/LRS ratio between age categories (4.93 ± 3.85 versus 8.44 ± 4.49, *P* = 0.009) disappeared within every group, and the negative peak current never differed by age category (full‐cohort *P* = 0.54). We then repeated the between‐group comparisons underlying the two detection steps within each age stratum (Table S16). Among younger patients, Step 1 separated control from pathological samples, with the negative peak current at 0.0267 ± 0.0038 versus 0.0157 ± 0.0058 A (*P* = 0.001, Cohen's *d* = −2.06), and Step 2 separated GBC from cholelithiasis, with the HRS/LRS ratio at 12.85 ± 0.40 versus 2.69 ± 0.16 (*P* = 0.009, *d* = +44.6). Among older patients, Step 1 persisted, with the negative peak current at 0.0283 ± 0.0017 versus 0.0080 ± 0.0044 A (*P* = 0.002, *d* = −5.55). Step 2 was preserved descriptively (the single older cholelithiasis patient's ratio of 2.71 versus 12.32 ± 1.36 in older GBC patients) but was not formally testable because only one cholelithiasis patient was ≥60 years old. Applying the bootstrap‐averaged cut‐off values (0.0224 A and 2.9232) within each age stratum reproduced the overall leave‐one‐out performance, with 29 of 30 patients correctly classified at Step 1 and 19 of 20 at Step 2. The two misclassified patients were precisely the boundary patients identified by the overall leave‐one‐out analysis (one control patient at Step 1 and one cholelithiasis patient at Step 2), both of whom fell in the younger stratum, which contained 17 of the 30 patients, indicating no age‐dependent concentration of errors. Consistent with the null within‐group correlations for continuous age (Tables S8 and S9), these categorical analyses indicate that the intergroup electrical differences are not attributable to age.

Finally, the between‐group effect sizes of the electrical signatures spanned Cohen's *d* = 4.06 to 17.09, with every 95% confidence interval excluding zero (Table S17). These values exceeded the largest between‐group clinical effect size, *d* = 1.73 for WBC count (control versus GBC, Table S18), by more than twofold at the lower end and by roughly an order of magnitude at the upper end. Together, these exploratory analyses indicate that the intergroup electrical differences are associated with disease state rather than with the demographic and clinical covariates examined, although the sample size (N = 30) precludes formal multivariate adjustment.

### 2.5 Cu^2^^+^/Zn^2^^+^ Modulation and Switching Mechanism

The physical architecture and signal transmission mechanism of a memristor bear a striking resemblance to those of a biological synapse [34, 35, 36, 37]. The processes of formation and rupture of conductive filaments (CFs) within memristors are analogous to the excitation and inhibition observed in biological synapses [38, 39]. The active electrode releases metal cations, which then migrate within the dielectric layer under the influence of an electric field and form conductive filaments through reduction reactions [40, 41, 42]. The transport of these metal cations in the dielectric layer resembles the accumulation and release of Ca^2^^+^ ions in the presynaptic and postsynaptic clefts of biological systems, playing a pivotal role in initiating plasticity changes [43]. Under the influence of an electric field, the inherently mobile cations within the dielectric layer engage in dynamic competitive migration with the metal cations released through the oxidation of the active electrode. This competitive process not only profoundly influences the formation kinetics, composition, and morphology of conductive filaments, but also finely regulates the resistance switching parameters of memristors at the microscopic level [44, 45]. This offers us a core theoretical framework for analyzing the resistive switching behavior of bile‐medium bio‐memristors. According to this framework, disease‐associated alteration in biliary ionic composition would be expected to perturb filament formation dynamics and thereby shift the switching parameters of the device.

Bile is an active secretory product of hepatocytes and biliary epithelial cells, and its composition therefore reflects the state of the hepatobiliary system [46]. Molecular profiling of clinical bile has revealed extensive differences among non‐disease, cholelithiasis, and GBC bile across proteins and metabolites, with increased inflammation‐related pathways in GBC, consistent with the release of tumor‐derived proteins and metabolites from malignant cells [47]. Among these biochemical alterations, the balance of copper and zinc is of particular interest. Clinical studies have consistently reported that serum and biliary copper levels rise while zinc levels fall as disease progresses from cholelithiasis to gallbladder carcinoma, producing a progressive increase in the Cu/Zn ratio [48, 49, 50, 51, 52]. Because Cu^2+^ and Zn^2+^ are redox‐active transition metal ions that participate directly in electrochemical processes at the device interface, we reasoned that disease‐associated changes in the biliary Cu/Zn balance could be transduced into measurable differences in memristive electrical response. Simultaneously, a large number of clinical and basic research have substantiated that the dysregulation of copper and zinc ion homeostasis plays a pivotal role in the pathogenesis and progression of hepatobiliary disorders [53, 54].

Therefore, we conducted a quantitative analysis of the copper and zinc ion content in the bile samples of the patients in this study. Quantitative ICP‐MS analysis of the thirty clinical bile samples revealed a monotonic gradient in the metal profile across the three groups (Table S19). Biliary Zn decreased from 5.54 mg/L (control) to 5.36 mg/L (cholelithiasis) to 3.48 mg/L (GBC), whereas biliary Cu increased from 4.48 to 4.73 to 6.53 mg/L, so the Cu/Zn ratio increased from 0.83 to 0.89 to 1.97 (Table S20). The GBC group differed significantly from both non‐malignant groups in Zn, Cu, and the Cu/Zn ratio (all *P* < 0.05, Welch *t*‐tests, *n* = 10 per group), matching the published values that motivated the study design [55]. At the patient level, the Cu/Zn ratio correlated negatively with the negative peak current (Spearman *ρ* = −0.87, *P* < 0.001) and positively with HRS/LRS (*ρ* = +0.68, *P* < 0.001); biliary Zn correlated positively with the negative peak current (*ρ* = +0.70, P < 0.001) and negatively with HRS/LRS (*ρ* = −0.63, *P* < 0.001); and biliary Cu showed weaker but directionally concordant associations (*ρ* = −0.53, *P* = 0.002 and *ρ* = +0.51, *P* = 0.004, respectively) (Table S21). These associations were largely retained within each disease group, indicating that the Cu/Zn signal operates at the patient level rather than being carried by the group means alone. A Monte Carlo power analysis using the published group parameters indicates that the group‐level Cu/Zn differences underlying the proposed mechanism lie within the resolving power of this study (power 62.0%–98.0% at *n* = 10 per group, Table S22).

**FIGURE 4:**
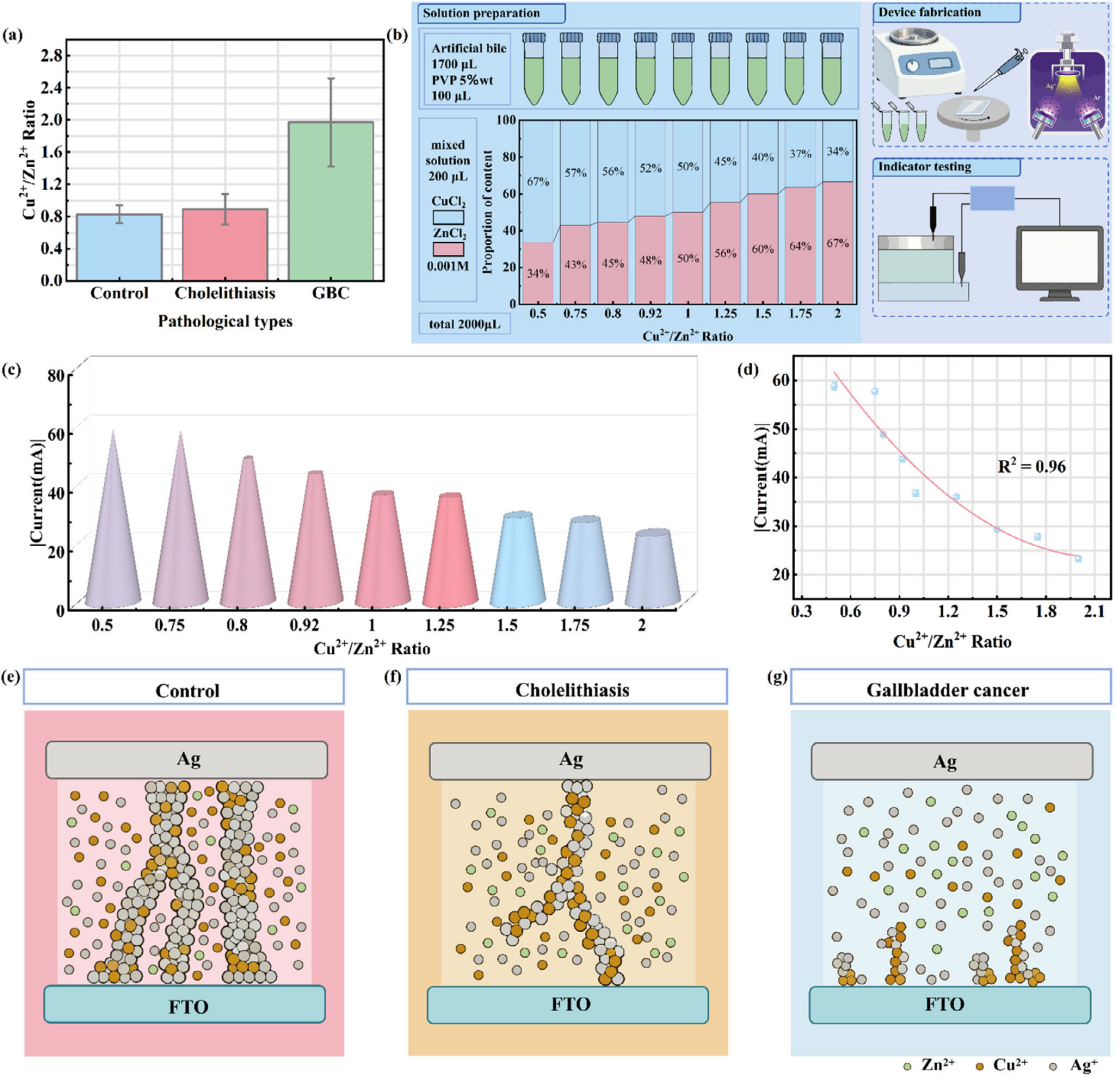
Mechanistic study of ion‐induced resistive switching in bile‐based memristors using artificial bile solutions. (a) Cu^2^^+^/Zn^2^^+^ ratios measured by ICP‐MS in clinical bile samples across control, cholelithiasis, and GBC groups. (b) Schematic workflow of artificial bile preparation with varying Cu^2^^+^/Zn^2^^+^ ratios, device fabrication, and electrical characterization. (c) |Current| responses of devices fabricated with artificial bile at different Cu^2^^+^/Zn^2^^+^ ratios (0.5–2). (d) Correlation between Cu^2^^+^/Zn^2^^+^ ratio and |Current|, showing a strong negative correlation (R^2^ = 0.96). (e–g) Schematic illustration of the proposed resistive switching mechanism in devices based on (e) control bile, (f) cholelithiasis bile, and (g) GBC bile, depicting the influence of Cu^2^^+^ and Zn^2^^+^ on Ag^+^ conductive filament formation.

To systematically investigate the mechanistic relationship between bile ion composition and memristive electrical response, we designed an integrated experimental framework combining clinical bile Cu/Zn quantification with artificial bile simulation experiments (Figure 4a). As a redox‐active transition metal ion, an elevated level of copper ions can catalyze the generation of reactive oxygen species (ROS), thereby inducing oxidative stress. This further activates inflammatory pathways such as NF‐κB, promotes the release of inflammatory mediators, and exacerbates the inflammatory response in the gallbladder wall [55, 56, 57, 58]. Zinc serves as a constituent of various antioxidant enzymes. A decrease in zinc levels impairs the body's antioxidant capacity, rendering cells more susceptible to ROS‐induced damage, consequently intensifying inflammatory reactions and tissue injury [59, 60]. Simultaneously, the disruption of copper and zinc homeostasis and the overproduction of ROS may result in DNA damage, protein modification, and potentially contribute to cancer development [61]. Currently, the copper‐to‐zinc ratio in bile has demonstrated considerable potential in the detection and prognostic assessment of biliary tract diseases. Several studies have indicated that the bile Cu/Zn ratio in patients with gallbladder carcinoma is significantly elevated compared to that in patients with chronic cholecystitis and control subjects. As shown in Figure 4b, we observed that the upward trend in the Cu/Zn ratio in the bile of the three populations was entirely consistent with the downward trend in the peak current of the corresponding memristors [48, 49, 50, 51, 52]. We hypothesize that the levels of Cu and Zn in bile may represent critical factors influencing the electrical properties of the devices, thereby enabling them to exhibit testing efficacy.

To validate this hypothesis, this study devised an artificial bile simulation experiment. Artificial bile typically comprises an aqueous solution of lecithin, bile acids, bile salts, and cholesterol, encompassing the pivotal constituents found in natural bile [62]. Based on artificial bile, we introduced copper salt and zinc salt solutions at varying concentration gradients and established Cu/Zn ratio gradients that encompassed the clinical detection range. Concurrently, we maintained a consistent total ionic strength across all experimental groups to mitigate non‐target interference (as depicted in Figure 4a). Following repeated electrical tests conducted using a testing protocol identical to that employed for clinical samples, we observed that as the Cu/Zn ratio in artificial bile increased in a gradient manner, the corresponding memristor exhibited a significant, gradient‐like decrease in both positive and negative scanning peak currents. This trend perfectly aligned with the electrical variations observed in clinical bile samples (as shown in Figure 4c). Critically, within the Cu/Zn ratio range corresponding to the three clinical bile groups, the device current response amplitude of the artificial bile system closely matched the measured range of clinical samples. Given that human bile contains various potential complexing agents, such as proteins and amino acids, they can form complexes with Cu^2^^+^ and Zn^2^^+^ that exhibit varying stabilities and charges, thereby influencing ion migration. Therefore, the device current response amplitude of artificial bile system is slightly different from the measured range of clinical samples. Correlation analysis further reveals a significant negative linear correlation between the Cu/Zn ratio in artificial bile and the peak current of the device (R^2^ ≥ 0.96) (as shown in Figure 4d). These results support a proposed model in which the Cu^2+^/Zn^2+^ ratio regulates the electrical response of the memristor, providing a mechanistic explanation for the device's ability to distinguish clinical bile samples from GBC patients. This provides a mechanistic explanation for the device's ability to distinguish clinical bile samples from GBC patients.

The proposed regulatory effect of the Cu/Zn ratio can be interpreted within the cation competitive migration theory discussed earlier. From a thermodynamic perspective, the standard electrode potential of Ag^+^/Ag is +0.799 V, which is higher than that of Cu^2^^+^/Cu (+0.34 V). Theoretically, Ag^+^ is more prone to reduction and deposition, leading to the formation of silver‐based conductive filaments [63]. However, when the concentration of Cu^2^^+^ in the system is elevated, Cu^2^^+^ will occupy the active sites on the electrode surface, thereby inhibiting the reduction deposition of Ag^+^. This results in a transition of the conductive filaments from being silver‐dominated to copper‐dominated (Figure 4e–g). Copper‐based conductive filaments are susceptible to irreversible dissolution and fracture under reverse bias conditions, making it challenging to maintain stable high‐current transport [64, 65, 66]. Simultaneously, according to the theory of electrochemical corrosion and passivation, Cu^2^^+^ readily reacts with water molecules or hydroxyl ions in aqueous media to form insulating passivation layers, such as copper hydroxide (Cu(OH)_2_) or copper oxide (CuO) [67]. These passivation layers form a physical barrier directly on the surface of the FTO electrode, increasing the interfacial resistance and consequently limiting the current enhancement [68, 69]. On the other hand, based on the ionic conductivity theory of electrolytes, the conductivity (κ) of a solution is positively correlated with the concentration (c_i_) of ions and their molar conductivity (λ_i_) (κ ∝ Σc_i_λ_i_) [70]. Zn^2^^+^ ions typically possess a relatively small hydrated radius and high mobility, rendering them effective charge carriers that enhance the conductivity of the solution.

### 2.6 Conduction Mechanisms of Bile‐Based Memristors

**FIGURE 5:**
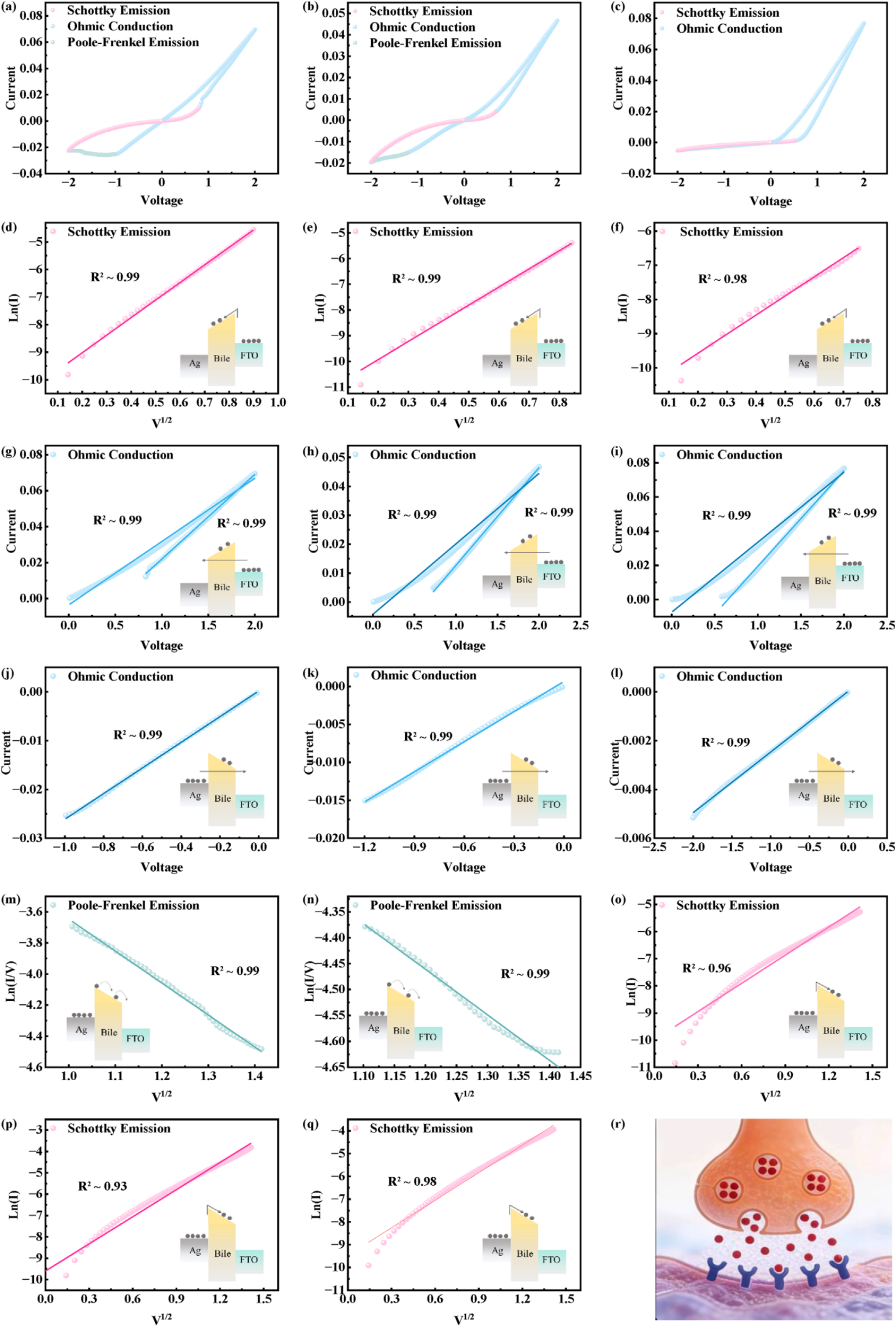
Conduction mechanism analysis of bile‐based memristors. (a–c) Typical I–V curves of memristors with different bile samples, indicating Schottky emission, ohmic conduction, and Poole‐Frenkel emission mechanisms. (d–f) Linear fitting of ln(I) versus V^1^/^2^ under positive bias, confirming Schottky emission behavior. (g–l) Linear fitting of I versus V in the low‐resistance state, verifying ohmic conduction characteristics. (m–n) Linear fitting of ln(I/V) versus V^1^/^2^ under negative bias, revealing Poole‐Frenkel emission. (o–q) Additional Schottky emission fitting for different pathological samples. (r) Schematic illustration of ion‐mediated conduction behavior at the device interface.

The electrical conduction mechanism of the Ag/bile/FTO memristor is grounded in the redox reactions and electric field‐directed migration of metal ions. The carrier transport mechanism encompasses ohmic conduction, Schottky emission, and Poole‐Frenkel (P‐F) emission. To elucidate the conduction mechanism, we performed a segmented fitting analysis on the I‐V curve obtained from a complete scanning cycle, with the piecewise characteristic fitting curves depicted in Figure 5a–c. Under a low forward bias, the extent of oxidative dissolution of the Ag top electrode remains extremely minimal, with no formation of conductive filaments (CFs) within the functional layer. Under such circumstances, carrier transport is governed by the Schottky barrier at the Ag/bile interface, primarily through hot electron emission, adhering to the Schottky emission mechanism (Figure 5d–f). When the forward bias surpasses the threshold voltage, metal ions undergo directional migration driven by the electric field, subsequently reducing and depositing onto the FTO bottom electrode, ultimately forming metallic CFs that bridge the two electrodes. The bulk resistance of the established CFs emerges as the predominant factor, resulting in a linear dependence between current and voltage, consistent with ohmic conduction (Figure 5g–i) [71, 72]. During the forward flyback process (from 2 to 0 V), the formed CFs did not completely rupture, and the *I–V* characteristics exhibited a strict linear dependence, demonstrating stable ohmic conduction behavior (Figure 5g–i). Under reverse low bias conditions, CFs underwent localized oxidation and dissolution while maintaining a continuous conductive pathway, with ohmic conduction remaining the predominant transport mechanism (Figure 5j–l). Under reverse high bias, CFs were completely oxidized and fractured, reverting the device to a high‐resistance state. Carrier transport was then regulated by the trap energy levels within the bulk phase of the bile functional layer, primarily involving trap‐assisted carrier excitation, consistent with the Poole‐Frenkel (P‐F) emission mechanism (Figure 5m,n) [73]. During the reverse flyback process (from −2 to 0 V), the electric field strength continuously decreases. At this juncture, carrier transport is once again dominated by the Schottky barrier at the FTO/bile interface, which conforms to the Schottky emission mechanism (Figure 5o–q).

The Ag/bile/FTO memristor adheres to a consistent electrical conduction framework across various bile states. Nevertheless, consistent with the proposed influence of the pathologically altered Cu/Zn ratio, the GBC group exhibits discernible differences from the control and cholelithiasis groups in terms of the transition threshold, characteristic interval, and conduction characteristics. During the forward scan interval, the Schottky emission ranges for the control, cholelithiasis, and GBC groups are 0–0.8 V, 0–0.7 V, and 0–0.57 V, respectively. The threshold voltage for mechanism transition progressively decreases as the Cu/Zn ratio increases. In the reverse voltage scanning range, both the control and cholelithiasis groups demonstrate ohmic conduction characteristics under low reverse bias conditions, transitioning to a typical Poole‐Frenkel (P‐F) emission‐dominated regime under high reverse bias. Conversely, the GBC group maintains only low‐conductance ohmic conduction throughout the full reverse bias range of 0–−2.0 V, lacking P‐F emission characteristics, thereby presenting a distinguishing feature from non‐cancerous gallbladders.

### 2.7 Memristor‐Based Circuitry for Real‐Time Detection

GBC is difficult to be diagnosed because of its occult onset and nonspecific symptoms. The current clinical detection methods are cumbersome and lack of timeliness, and the sensitivity for identifying gallbladder malignancy remains insufficient [1, 74, 75, 76, 77, 78]. In this proof‐of‐concept study, we developed an Ag/Bile/FTO memristor sensing unit and demonstrated that its resistive switching behavior differs systematically across bile samples from patients with GBC, cholelithiasis, and controls. Integration of this memristor with a logic operation circuit and a wireless transmission module could enable future development of a rapid detection system. We also present a conceptual design for potential integration of such a system with the ERCP platform.

**FIGURE 6:**
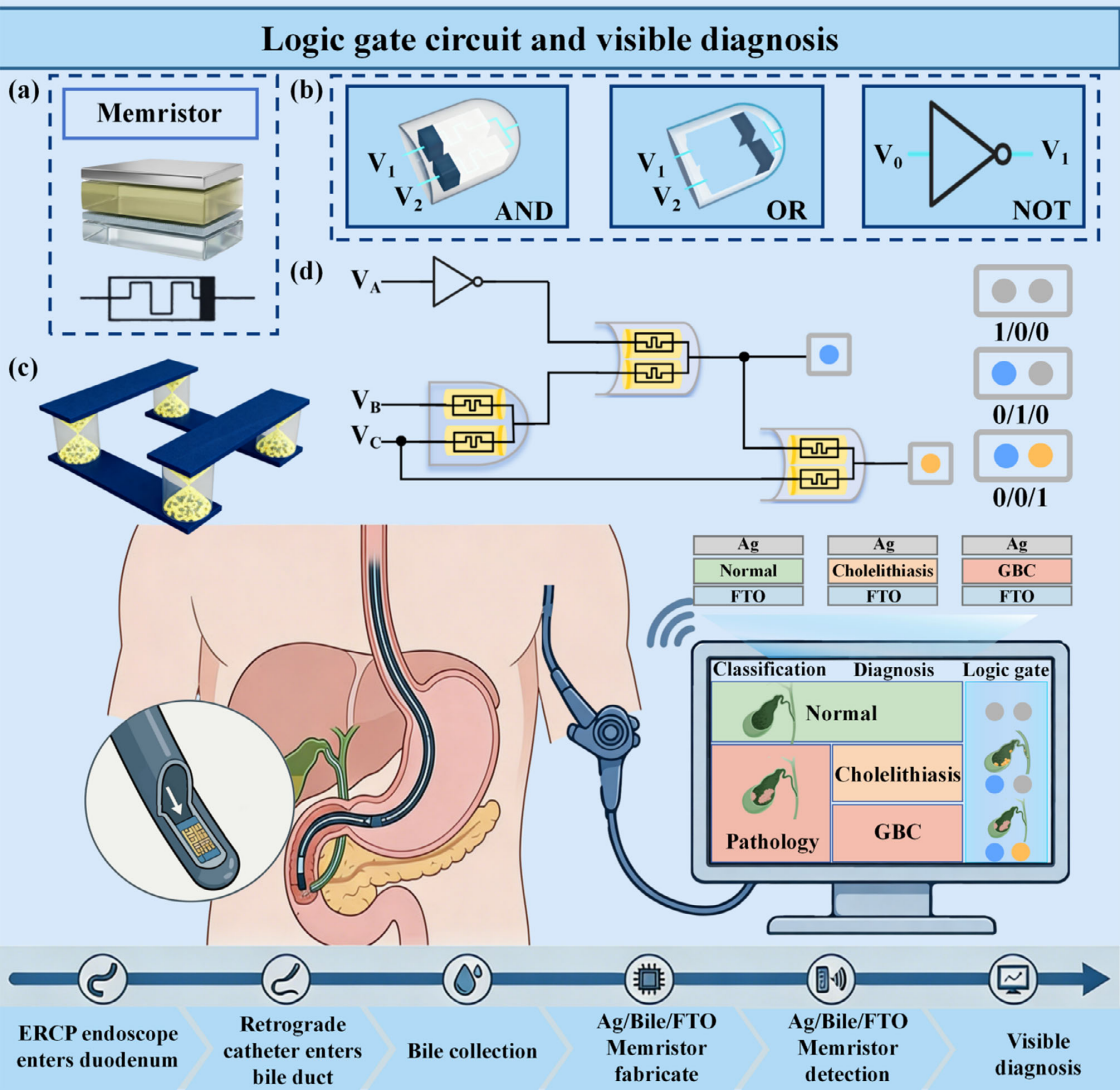
Conceptual memristive logic gates and the ERCP‐integrated visual detection system. (a) Schematic of the Ag/bile/FTO memristor. (b) Basic Boolean logic operations (AND, OR, NOT) designed based on memristive resistive switching. (c) Hierarchical logic circuit constructed by cascading memristive logic gates for multi‐class sample classification. (d) Schematic workflow of the ERCP‐integrated detection platform, including in situ bile sampling, memristive detection, logic‐based classification, and real‐time visual output for gallbladder disease detection.

In this study, we constructed a 2 × 2 memristor arrays as the core structure of the detection unit (Figure 6c). A uniform standard input voltage of 2.0 V was applied to the array, and electrical performance simulation tests were conducted using the Simulink module. The results indicated that the device's output current corresponding to control bile was 360 mA, which decreased to 80 mA for cholelithiasis bile, and further dropped to only 32 mA for GBC bile (Figure S11). The three sample types exhibited gradient current output characteristics, providing a stable and quantifiable electrical foundation for subsequent signal differentiation.

Based on the two core markers of negative peak current and HRS/LRS switching ratio, we leveraged the resistive switching characteristics of memristors to construct AND, OR, and NOT basic Boolean logic gates (Figure 6b). Logic 0 and logic 1 are defined by the high‐resistance state and low‐resistance state, and the core logic operation is realized through the device series parallel structure and reverse resistance transformer response (Figure S12). The output characteristics of these logic gates, as evaluated by Matlab simulations, were consistent with the design expectations (Figure S13). Building on this foundation, we designed a hierarchical detection logic circuit by cascading these three types of logic gates (Figure S14). Subsequently, using the two types of electrical characteristics as input signals, we mapped the electrical responses of different pathological states into three distinguishable output states through multilevel logic operations (Figure 6d). Simulink simulations further indicated that the designed multilevel logic circuit can correctly realize the expected classification mapping (Figure S15). In principle, this circuit design would enable unsupervised automatic classification of three sample types without requiring additional algorithmic processing, thereby providing a conceptual basis for hardware‐level support for the direct output of detection results.

To facilitate clinical translation, this study further integrated the memristor sensing unit with ERCP equipment to construct a conceptual visual detection system. A conceptual framework for integrating this detection strategy with ERCP is presented in Figure 6. The entire process commences with an endoscopic transoral approach and the retrograde insertion of a catheter into the bile duct. Subsequently, trace bile samples would be collected in situ via the catheter and injected through the inlet port of a single‐use disposable cartridge. The cartridge is a sealed microfluidic chip whose empty interior chamber, of a few microliters, is bounded on one side by a planar FTO electrode and on the other by a pre‐patterned Ag electrode, the two electrode faces separated by a micrometer‐scale gap that is fixed at manufacture so that the ±2.0 V sweep validated in this study remains sufficient for switching and an injected drop of bile is drawn into the gap by capillary action; a vent outlet allows displaced air to escape during filling. The patient's freshly collected bile would fill the inter‐electrode gap and thereby form the Ag/bile/FTO junction at the point of care, without spin coating, annealing, or sputtering. The cartridge would then be seated in a portable reader that applies the same voltage sweep protocol as the laboratory measurement and performs the two‐step classification. The detection signals are then input into a hierarchical detection logic circuit for automatic classification computation. Finally, in the conceptual workflow, the results would be transmitted wirelessly and fed back to the terminal interface, where visual differentiation among the three sample types could be achieved through color coding and textual annotations, providing the operator with a conceptual basis for real‐time discrimination.

To position the detection performance of the memristor platform, we summarized the published point estimates of conventional diagnostic approaches, including serum CA19‐9, ultrasound‐based methods, cross‐sectional imaging, and MRCP (Table S23). In the published literature, the reported sensitivities of these approaches range from 51.6% (ultrasound) to 92.5% (MRCP) and the specificities from 74.3% to 92%. In this cohort, CA19‐9 was recorded for all ten GBC patients and exceeded 37 U/mL in 7 of 10 (70%, exact 95% CI 34.8–93.3%), consistent with the published meta‐analytic sensitivity of about 70%. The three CA19‐9‐negative patients, one pT1bN0M0, one T2N0M0, and one T3N1M0 case, were all correctly classified by the memristor platform at Step 2 (McNemar exact *P* = 0.25 for the paired comparison across the ten patients). The memristor platform discriminated GBC from non‐GBC bile samples with a sensitivity of 100%, a specificity of 95% (19/20), and an accuracy of 96.7% under patient‐level leave‐one‐out cross‐validation, numerically above every conventional point estimate listed in Table S23. Due to the majority of values in the control group and cholelithiasis group being too low and below the detection limit of the instrument, specific numerical values are not available. Thus, no inter group statistical comparison was conducted. However, relevant cohort information is still presented in Table S25. Because the sources differ in study design, cohort, sample size, and clinical setting, and because the confidence intervals of our estimates remain wide at this sample size, this exploratory side‐by‐side summary should not be interpreted as evidence of clinical superiority, and formal non‐inferiority or superiority testing in larger cohorts is required before any comparative conclusion can be drawn [79, 80, 81].

Several limitations should be acknowledged. First, the three groups differed in demographic and clinical characteristics, including age, gender, bilirubin, liver enzymes, and inflammatory indices. Because age and gender can affect bile composition, the observed differences in memristive response could be partly attributable to demographic variation rather than disease‐specific alterations, and the sample size (N = 30) precludes formal multivariate adjustment. The inclusion criterion of serum CA19‐9 below 37 U/mL for control participants, adopted to reduce the probability of undiagnosed malignancy among controls, introduces additional selection bias that may inflate apparent group separation. Moreover, only the CA19‐9 values of the GBC group were recorded completely. Due to the majority of values in the control group and the cholelithiasis group being too low and below the detection limit of the instrument, specific numerical values cannot be provided Therefore, it is impossible to perform cohort‐specific estimation for this biomarker. Second, the Cu^2+^/Zn^2+^ transduction mechanism is presented as a proposed model rather than a verified mechanism. The model is supported by experiments in a simplified artificial bile system and by clinical observations of an elevated Cu/Zn ratio in gallbladder carcinoma [48, 49, 50], but whether these trace element alterations directly drive the differential memristive response requires functional verification of native bile. In addition, bile contains numerous other constituents that may also influence the memristive response, including bile acids, bilirubin, proteins, lipids, and inflammatory mediators. The relative contributions of these components to the observed electrical signatures were not systematically examined in this study and warrant investigation in future work. In addition, exfoliated malignant cells and specific cytokines were not directly quantified in this prospective cohort, and parallel bile cytology and cytokine profiling in a prospective cohort represent an important direction for future work. Last, the Boolean logic circuit was evaluated through simulation, and the ERCP‐integrated detection platform remains a conceptual design that has not been physically constructed or clinically tested.

## 3 Conclusions

In conclusion, we have developed an Ag/Bile/FTO memristor using clinically sourced bile samples and characterized its stable resistive switching behavior. The memristive electrical signatures, particularly negative peak current and the HRS/LRS ratio, differ systematically across controls, cholelithiasis patients, and GBC patients, enabling a two‐step detection strategy with encouraging performance in this pilot cohort (N = 30). A conceptual ERCP‐integrated sensing platform was also designed to illustrate a potential future integration pathway. These findings establish the technical feasibility of memristor‐based bile detection and provide a foundation for developing rapid detection technology that is expected to complement existing clinical approaches to improve gallbladder cancer identification.

## 4 Materials and Methods

### 4.1 Study Design and Participants

This study was approved by the Ethics Committee of the First Affiliated Hospital of Xi'an Jiaotong University (approval number: XJTU1AF2026LSYY‐0103) and conducted in accordance with the Declaration of Helsinki. Written informed consent was obtained from all participants prior to sample collection. Bile samples were collected from patients undergoing ERCP or hepatobiliary surgery and assigned to one of three groups based on final clinical and pathological diagnosis:

Control group. Patients undergoing hemihepatectomy for indications not involving the biliary system (hepatic hemangioma), in whom intraoperative examination confirmed the absence of gallbladder and biliary tract abnormalities. Additional eligibility requirements were: (i) No evidence of gallstones, gallbladder polyps, or gallbladder wall thickening (> 3 mm) on preoperative abdominal ultrasound, CT, or Magnetic Resonance Cholangiopancreatography (MRCP). (ii) Liver function parameters (ALT, AST, ALP, GGT, total bilirubin, direct bilirubin) within the institutional normal reference range. (iii) Serum CA19‐9 < 37 U/mL. (iv) No prior history of gallstone disease or cholecystectomy. The liver function values were measured on the first day after surgery.

Cholelithiasis group. Patients with radiologically confirmed gallbladder stones (ultrasound and/or CT and/or MRCP) who underwent elective cholecystectomy. Postoperative histopathology confirmed chronic cholecystitis without evidence of intraepithelial neoplasia or invasive carcinoma. Complete liver function tests and serum CA19‐9 results were required.

Gallbladder cancer (GBC) group. The patients were highly suspected of gallbladder cancer during the intraoperative exploration, and it was difficult to identify it on the preoperative imaging. All diagnoses were confirmed by postoperative histopathology demonstrating primary gallbladder adenocarcinoma (any histological subtype). Tumor stage was classified according to the American Joint Committee on Cancer (AJCC) 8th edition TNM staging system.

Exclusion criteria applied uniformly to all three groups were: (i) Concurrent hepatobiliary malignancy other than GBC, including hepatocellular carcinoma, cholangiocarcinoma, and ampullary carcinoma. (ii) Prior chemotherapy, radiotherapy, targeted therapy, or immunotherapy. (iii) Acute cholangitis (Tokyo Guidelines 2018 criteria), acute pancreatitis (revised Atlanta classification), or liver abscess. (iv) Use of metal ion‐containing medications, including copper supplements, zinc preparations, and bismuth‐containing agents, within 30 days before sample collection. (v) Severe hepatic or renal impairment (Child–Pugh class C cirrhosis estimated glomerular filtration rate < 30 mL/min/1.73 m^2^). (vi) Wilson disease, primary biliary cholangitis, or primary sclerosing cholangitis. (vii) Prior biliary surgery (cholecystostomy, choledochojejunostomy, or bilioenteric anastomosis). (viii) Coagulopathy (international normalized ratio > 1.5 or platelet count < 50 × 10^9^/L). (ix) Pregnancy or lactation. (x) Macroscopically contaminated bile samples, exhibiting visible hemobilia or purulent bile. (xi) The patient was younger than 18 years old. In this study, a total of 30 patients were enrolled, with 10 patients in each group.

### 4.2 Sample Collection Procedure

Bile samples (2–5 mL) were collected intraoperatively under sterile conditions by two board‐certified attending surgeons using dedicated sterile, DNase/RNase‐free polypropylene containers. For the control group, bile was aspirated from the gallbladder in situ immediately after cholecystectomy, with intraoperative confirmation of biliary normality. In both the cholelithiasis group and the gallbladder cancer group, bile samples were collected immediately following cholecystectomy and prior to specimen fixation. Within 30 min of collection, each sample underwent on‐site quality control: macroscopic inspection (golden‐yellow color, transparent clarity) and pH verification (7.0–8.5, calibrated microelectrode). Samples exhibiting visible hemobilia or purulent contamination were excluded and documented in a pre‐specified sub‐group log. Samples passing QC are immediately placed on wet ice and transported. After full and uniform oscillation, move to the refrigerator at ‐80 °C for freezing. The time from intraoperative collection to freezing did not exceed 30 min. Spare parts shall be reserved during the manufacture of the device, so that the measurement may be repeated. Throughout the study, all fabrication batches included at least one sample from each of the three clinical groups processed on the same day to minimize batch effects.

### 4.3 Sample Storage Conditions

All bile samples were stored at −80°C. Storage freezers were equipped with continuous temperature monitoring and alarm systems (set point −80°C ± 5°C), with maintenance logs reviewed weekly. Sample collection dates were recorded for all specimens to enable post‐hoc analysis of storage duration as a potential confounder.

### 4.4 Device Fabrication

This study utilized clinically collected bile samples from patients with control bile, cholelithiasis, and GBC. The device was prepared using an Ag/bile/FTO sandwich structure. First, the FTO conductive glass was ultrasonically cleaned with deionized water, ethanol, and acetone (10 min each), and then dried under nitrogen for future use. Bile samples with different pathological states were uniformly spin‐coated onto the FTO substrate through a two‐step spin‐coating process (S1: 500 rpm, spin‐coating time 10 s. S2: 2,500 rpm, spin‐coating time 30 s). After spin‐coating, the samples were annealed at 30°C for 10 min to densify the bile functional layer. A magnetron sputtering device was used to deposit an Ag top electrode on the surface of the bile thin film, with a sputtering power of 80 W and a deposition time of 10 min.

### 4.5 Characterization of Memristor

The surface and cross‐sectional morphologies of the devices were characterized using scanning electron microscopy, and the elemental composition of the bile film was obtained through energy dispersive spectroscopy analysis. X‐ray diffraction and X‐ray photoelectron spectroscopy were employed to analyze the crystal structure and chemical state differences of different pathological bile functional layers, revealing the regulation mechanism of composition and structure on resistance switching behavior. Atomic force microscopy (AFM) characterizations were performed on a Bruker Dimension Icon AFM (Bruker, Germany). All bile thin‐film samples were scanned under ambient air conditions using ScanAsyst mode. The scan area was set as 2.0 × 2.0 µm for each specimen. Quantitative surface roughness parameters including average roughness (Ra) and root‐mean‐square roughness (Rq) were automatically extracted from the height topography images via NanoScope Analysis software.

A semiconductor parameter analyzer was used to test the electrical performance of the memristor, with a voltage scanning range of 0 V → 2.0 V → ‐2.0 V → 0 V and a scanning step size of 0.05 V. All tests were conducted in a room temperature atmospheric environment, with a continuous switching cycle test set to 500 cycles. The read voltages were 0.39 V, 0.46 V, and 0.41 V, respectively. The resistance values in the high and low resistance states were extracted from the tests, and the switching ratio was calculated. Gaussian distribution statistical analysis was used to assess the consistency of resistance states. The coefficient of variation was used to quantitatively evaluate the uniformity of SET voltage distribution, with each group of samples tested containing no fewer than 30 devices to ensure statistical validity.

### 4.6 Artificial Bile Preparation and Ion Ratio Adjustment

To verify the ion regulation mechanism, an artificial bile system containing bile acid, lecithin, cholesterol, and PVP was prepared, and different volumes of 0.001 M CuCl_2_ and ZnCl_2_ solutions were added to adjust the Cu^2^^+^/Zn^2^^+^ ratio gradient. The artificial bile‐based device underwent the same testing process as clinical samples, and the correlation between peak current and ion ratio was analyzed.

### 4.7 Quantification of Biliary Copper and Zinc

Biliary Cu and Zn were quantified by inductively coupled plasma mass spectrometry (ICP‐MS, PerkinElmer NexION 300D) operated in standard mode, monitoring ^6^ ^3^Cu and ^6^ ^6^Zn with ^10^ ^3^Rh as the internal standard. Bile samples were diluted with 2% (v/v) nitric acid, filtered through a 0.45 µm membrane, and measured against external calibration curves (0.2–500 µg/L, linear‐through‐zero regression, *r* = 0.9998 for Cu and 0.9994 for Zn). Quality control included a procedural blank below 1% of the lowest sample reading, internal‐standard recovery within the 80%–120% acceptance window, and back‐calculation of calibration standards within 10% of nominal values. All sample readings (12.8–110 µg/L) were at least tenfold above the practical quantification limit of approximately 1 µg/L.

### 4.8 Statistical Analysis

All primary analyses were performed at the patient level, treating the 90 devices as technical replicates of 30 patients (three per patient; 10 per group). The three device‐level measurements per patient (negative peak current, *I* ^−^; HRS/LRS ratio) were aggregated by the mean (median in a supplementary robustness analysis). Device‐level measurements were reported only as analytical repeatability (within‐patient CV%). Device‐level distributional analyses are in Section 4.5. Three‐group comparisons used the Kruskal–Wallis test with Dunn's post‐hoc test. Two‐group comparisons used two‐tailed Mann–Whitney U tests. Categorical variables were compared with Fisher's exact test. And biliary Cu, Zn, and Cu/Zn ratios with Welch's t‐tests (n = 10 per group). Two‐sided P < 0.05 was considered statistically significant. Demographic/clinical associations with the two core signatures were assessed by Spearman correlations (full cohort and within groups) and by partial correlations controlling for age or gender. The detection‐step comparisons were repeated within each gender with the bootstrap‐averaged cut‐offs re‐applied. Candidate signatures were screened by accuracy, Cohen's kappa, MCC, macro‐F1, and macro‐AUC (Figure 3a) and quantified with Cohen's *f* effect sizes and associated statistical power. Pairwise effect sizes were expressed as Cohen's *d* (pooled SD; 95% CIs from the Hedges' *g* standard error). Post‐hoc power was computed with GPower 3.1 (t‐test family, two‐tailed, α = 0.05) as a conservative approximation for non‐parametric comparisons, supplemented by Monte Carlo power analysis (Cu/Zn differences) and power curves against the effect size required for 80% power. Classification proceeded in two steps: Step 1 separated control from pathological patients using *I* ^−^, and Step 2 separated cholelithiasis from GBC using the HRS/LRS ratio. Each cut‐off maximized Youden's *J* index (*J* = sensitivity + specificity − 1) strictly within the training set of each fold or bootstrap iteration. Point estimates of sensitivity, specificity, PPV/NPV, accuracy, and AUC were obtained by leave‐one‐patient‐out cross‐validation (LOOCV; 30 folds for Step 1, 20 for Step 2), each fold holding out one patient with the cut‐off determined from the remaining patients only. Two‐step predictions were combined into a three‐class confusion matrix for overall accuracy. Patient‐level ROC curves were obtained for both steps. Sampling uncertainty was quantified by 10,000 iterations of group‐stratified patient‐level bootstrap (10 patients drawn with replacement per group per iteration, shared across the two steps). Cut‐offs were re‐estimated on the drawn patients and performance was evaluated strictly on out‐of‐bag patients (seeds 42 + b). The 95% confidence intervals were the 2.5th–97.5th percentiles of the bootstrap distributions (percentile method), reported alongside the LOOCV point estimates.

All analyses were implemented in Python 3.12 using NumPy, pandas, SciPy, and scikit‐learn. Figures were prepared with OriginPro 2024 and Python (Matplotlib). Post‐hoc power analysis was performed with GPower 3.1.

## Author Contributions

**Junming Zhu**: conceptualization, methodology, writing – original draft. **Yiyin Rong**: software, data curation, writing – original draft. **Bai Sun**: supervision, funding acquisition, writing – review and editing. **Ziwen Sun**: investigation. **Yifan Wang**: validation. **Xiaobin Ren**: formal analysis. **Zelin Cao**: visualization. **Kaikai Gao**: resources. **Jiawei Yu**: software. **Zhenkun Wang**: validation. **Haoxuan Ma**: visualization. **Jichun Guo**: formal analysis. **Kai Cao**: software. **Mengrun Kang**: resources. **Songling Wang**: formal analysis, supervision. **Fenggang Ren**: visualization. **Yi Lv**: visualization. **Qiang Lu**: project administration, supervision.

## Funding

This work was supported by the National Natural Science Foundation of China (52375575), the Science and Technology Plan Project of Shaanxi (2025JC‐TBZC‐05), the School Recruitment Joint Special Project of Shaanxi (SXXZGY202401), and the Basic Research Project of Xi'an Jiaotong University (xtr072024022, xtr062025002, and xtr052025010).

## Ethics Statement

This study was conducted in accordance with the ethical principles of the Declaration of Helsinki and was approved by the Ethics Committee of the First Affiliated Hospital of Xi'an Jiaotong University (XJTU1AF2026LSYY‐0103, approved on 22 January 2026). Bile samples were collected after ethical approval had been obtained, and written informed consent was obtained from all participants prior to sample collection. All patients provided written informed consent for the publication of their de‐identified clinical data.

## Clinical Trial Registration

This study does not involve a clinical trial as defined by the International Committee of Medical Journal Editors (ICMJE) or the WHO, and therefore clinical trial registration was not required.

## Conflicts of Interest

The authors declare no conflicts of interest.

## Supporting information




**Supporting File**: advs77893‐sup‐0001‐SuppMat.docx.

## Data Availability

The data that support the findings of this study are available from the corresponding author upon reasonable request, with individual patient‐level data provided in Table S25 of the Supporting Information.
