# Supplementary material for: Bio‐Memristor Based on Clinical Bile Samples: A Proof‐of‐Concept Pilot Study for Detection of Gallbladder Cancer
Source: Adv Sci (Weinh). 2026 Sep 27:e77893. Online ahead of print. doi: 10.1002/advs.77893 (PMC13616236; doi:10.1002/advs.77893)
Supplement: Supplementary file 1 [file ADVS-9999-e77893-s001.docx]

**Supporting Information**

**Bio-Memristor Based on Clinical Bile Samples: A Proof-of-Concept Pilot Study for Detection of Gallbladder Cancer**

*Junming Zhu, Yiyin Rong, Bai Sun, Ziwen Sun, Yifan Wang, Xiaobin Ren, Zelin Cao, Kaikai Gao, Jiawei Yu, Zhenkun Wang, Haoxuan Ma, Jichun Guo, Kai Cao, Mengrun Kang, Songling Wang, Fenggang Ren, Yi Lv, and Qiang Lu*

J. Zhu, Y. Rong, Prof. B. Sun, Z. Sun, Y. Wang, J. Yu, Z. Wang, H. Ma, J. Guo, K. Cao, M. Kang, Dr. Q. Lu

Department of Abdominal Surgery, The First Affiliated Hospital of Xi'an Jiaotong University, Xi'an, Shaanxi, 710061, China

Email: baisun@xjtu.edu.cn (B. Sun); [luqiang2020@xjtufh.edu.cn](mailto:luqiang2020@xjtufh.edu.cn) (Q. Lu)

Dr. Q. Lu, Dr. F. Ren, Prof. Y. Lv

National Local Joint Engineering Research Center for Precision Surgery and Regenerative Medicine, The First Affiliated Hospital of Xi'an Jiaotong University, Xi'an, Shaanxi, 710061

X. Ren, Z. Cao, K. Gao, Prof. B. Sun

Frontier Institute of Science and Technology, and Interdisciplinary Research Center of Frontier Science and Technology, Xi'an Jiaotong University, Xi'an, Shaanxi 710049, China

Micro-and Nano-technology Research Center, State Key Laboratory for Manufacturing Systems Engineering, Xi'an Jiaotong University, Xi'an, Shaanxi 710049

Prof. S. L. Wang

State Key Laboratory of Structural Chemistry, Fujian Institute of Research on the Structure of Matter, Chinese Academy of Sciences, Fuzhou 350002, China

(a)

(b)

(c)

(d)

(e)

(f)

(g)

(h)

(i)

(j)

(k)

(l)

**Figure S1.** EDS elemental mapping of bile-based memristors. (a–f) Surface elemental distribution of C, N, O, P, Na, and Cl. (g–l) Cross-sectional elemental mapping showing the layered distribution of key elements in the device.

**Figure S2.** Comprehensive morphological evaluation and statistical assessment of fabrication reproducibility across five batches. (a) Representative cross-sectional images of the multi-layered devices. (b-f) Statistical film thickness distribution (Device_ID 1–6). Annotated values indicate the average thickness ranging narrowly between ~195 and 211 nm. (g–k) Surface roughness evaluation ($R_{a}$ and RMS) derived from ImageJ grayscale analysis of planar SEM images. (l–n) Overall statistical histograms with Gaussian fitting for thickness, $R_{a}$, and RMS, showing mean values ($\mu$) and coefficients of variation (CV) of $\mu=202.23$ nm ($CV=0.031$), $\mu=17.58$ nm ($CV=0.053$), and $\mu=19.37$ nm ($CV=0.062$), respectively. (o) Trends of average parameters across batches, confirming process stability. (p) Representative 2D AFM height sensor maps (2.0 µm scan area). (q–u) Summary of the precise AFM-measured surface roughness.

**Figure S3**. X-ray photoelectron spectroscopy (XPS) spectra of elements in cholelithiasis samples. (a) Na 1s XPS spectrum of cholelithiasis sample, with experimental data, background, and fitting curve. (b) C 1s XPS spectrum of cholelithiasis sample, showing peaks of different carbon-containing functional groups, along with experimental data, background, and fitting curve. (c) Cl 2p XPS spectrum of cholelithiasis sample, displaying spin - orbit splitting peaks, with experimental data, background, and fitting curve. (d) Ca 2p XPS spectrum of cholelithiasis sample, depicting calcium characteristic peaks, along with experimental data, background, and fitting curve.

**Figure S4**. X-ray photoelectron spectroscopy (XPS) spectra of elements in gallbladder cancer samples. (a) Na 1s XPS spectrum of gallbladder cancer sample, with experimental data, background, and fitting curve. (b) C 1s XPS spectrum of gallbladder cancer sample, including peaks of various carbon-related species, along with experimental data, background, and fitting curve. (c) Cl 2p XPS spectrum of gallbladder cancer sample, showing spin - orbit splitting, with experimental data, background, and fitting curve. (d) Ca 2p XPS spectrum of gallbladder cancer sample, depicting calcium characteristic peaks, along with experimental data, background, and fitting curve.

**Figure S5**. Electrical characteristics of the device in pure water and saline environments. (a) Current - voltage (I - V) curves for the first cycle in pure water (upper) and saline (lower). (b) Controlized resistance (Rₙ/Rₘ) as a function of cycling number in pure water (upper) and saline (lower). (c) Resistance variation with cycling number in pure water (upper) and saline (lower).

**Figure S6.** I–V overlay plots of 30 Ag/Control bile/FTO memristors, each measured over 50 consecutive cycles.

**Figure S7.** I–V overlay plots of 30 Ag/Cholelithiasis bile/FTO memristors, each measured over 50 consecutive cycles.

**Figure S8.** I–V overlay plots of 30 Ag/GBC bile/FTO memristors, each measured over 50 consecutive cycles.

**Figure S9**. Statistical evaluation and classification performance of electrical parameters for the discrimination of control, cholelithiasis, and GBC samples. (a-b) Bar charts illustrating the Cohen's f effect sizes (left y-axis) and the corresponding statistical power (right y-axis) for various electrical parameters at the (a) device-level and (b) patient-level. The specific Cohen's f values and an exceptionally high statistical power (>0.9999) are annotated above the bars. (c) Scatter plot combined with marginal density distributions for the Control, Cholelithiasis, and GBC groups, plotted against Current (mA) and HRS/LRS Ratio. Dashed lines indicate the optimal classification thresholds (|Current| = 0.0224 A and HRS/LRS Ratio = 2.9232). (d) Confusion matrix of the three-class classification model, exhibiting high diagnostic accuracy with 10 out of 10 GBC samples correctly identified. (e) Power curves as a function of Cohen's d for the classification models at different steps. The horizontal dashed line indicates the threshold for 80% statistical power. (f) Comparison of the observed Cohen's d effect sizes and the ratio of observed d to the d required for 80% power across different analytical steps.

**Figure S10**. Multi-step cross-validation and Bootstrap reliability evaluation of the electrical feature-based diagnostic model. (a-c) Frequency distribution histograms and Gaussian fitting curves of Current and HRS/LRS ratios across three sample states, annotated with mean and CV. (d, f) Bar charts of performance metrics for two-step classification tasks, presented as mean and SD calculated from 10,000 Bootstrap resampling iterations. The charts comprehensively report Sensitivity, Specificity, PPV, NPV, Accuracy, AUC, as well as the Cutoff value determined based on the Youden index maximization principle, along with the corresponding Youden index. (e, g) Confusion matrices for two-step binary classification based on the Leave-One-Patient-Out Cross-Validation (LPOCV) strategy. (h) Total confusion matrix summarizing both steps, demonstrating overall discriminative ability across Control, Cholelithiasis, and GBC. (i) Forest plot of evaluation metrics, presenting point estimates and 95% confidence intervals (95% CI) derived via Bootstrap. (j, k) ROC curves for the two-step cross-validation tasks, with panel (k) annotating the performance boundary corresponding to the Cutoff value determined by the Youden index.

(a)

(b)

**Figure S11**. Memristor array circuit simulation and current response characteristics. (a) Schematic of the 2×2 memristor array circuit constructed for bile sample detection. (b) Simulated output current of devices with control, cholelithiasis, and gallbladder cancer bile samples under a uniform 2.0 V input, showing gradient current distribution.

(b)

(a)

**Figure S12**. Schematic diagram of simulation circuits for logic OR gates (a) and logic AND gates (b) constructed using Ag/Bile/FTO memristors.

**Figure S13**. (a) OR logic circuit constructed with memristors. (b) Truth table of OR logic gate. (c) Simulation results of OR logic circuit. (d) AND logic circuit constructed with memristors. (e) Truth table of AND logic gate. (f) Simulation results of AND logic circuit.

**Figure S14**. Memristor-based logic circuits.

**(a)**

**(b)**

| **Control** | **Cholelithiasis** | **GBC** | **Yellow light** | **Blue light** |
| --- | --- | --- | --- | --- |
| 1 | 0 | 0 | 0 | 0 |
| 0 | 1 | 0 | 1 | 0 |
| 0 | 0 | 1 | 1 | 1 |

**Figure S15.** Logic operation results and truth table of the diagnostic circuit. (a) Output voltage responses corresponding to three diagnostic states: 1/0/0 (control), 0/1/0 (cholelithiasis), and 0/0/1 (gallbladder cancer). (b) Truth table mapping input signals (Control, Cholelithiasis, GBC) to output states (Yellow light, Blue light) for visual diagnosis.

**Figure S16.** Comparison of diagnostic performance between the proposed memristor platform and conventional clinical assays for gallbladder lesion identification. (a) Scatter plot of sensitivity versus specificity for all detection methods. The star represents the memristor system, the only strategy with both metrics above the 90% thresholds (dashed lines). (b) Bar graphs summarizing specificity (purple) and sensitivity (green) of serum CA19-9, US, CEUS, CECT, MRI, MRCP, and this memristor-based method, which achieves 95% specificity and 100% sensitivity.

**Table S1.** XRD results of control bile memristor

| 2θ(°) | Intensity | FWHM | Interplanar spacing (nm) | Scherrer width (nm) |
| --- | --- | --- | --- | --- |
| 26.520 | 9889.648 | 0.280 | 0.3358 | 29.1496 |
| 37.760 | 8817.516 | 0.320 | 0.2381 | 26.2375 |
| 51.520 | 7894.083 | 0.300 | 0.1772 | 29.4030 |
| 33.720 | 4234.871 | 0.280 | 0.2656 | 29.6468 |
| 65.520 | 3387.546 | 0.440 | 0.1424 | 21.4701 |
| 61.560 | 1557.490 | 0.420 | 0.1505 | 22.0162 |
| 26.520 | 9889.648 | 0.280 | 0.3358 | 29.1496 |
| 37.760 | 8817.516 | 0.320 | 0.2381 | 26.2375 |

X-ray wavelength used by Scheler formula and Bragg formula: 1.54060 Å

**Table S2.** XRD results of cholelithiasis bile memristor

| 2θ(°) | Intensity | FWHM | Interplanar spacing (nm) | Scherrer width (nm) |
| --- | --- | --- | --- | --- |
| 37.800 | 11320.511 | 0.580 | 0.2378 | 14.4776 |
| 26.500 | 11150.854 | 0.260 | 0.3361 | 31.3906 |
| 31.720 | 9167.658 | 0.200 | 0.2819 | 41.2934 |
| 51.560 | 6105.805 | 0.320 | 0.1771 | 27.5699 |
| 65.600 | 4424.898 | 0.420 | 0.1422 | 22.5026 |
| 33.740 | 2476.113 | 0.280 | 0.2654 | 29.6484 |
| 44.300 | 1857.173 | 0.360 | 0.2043 | 23.8258 |
| 61.620 | 1786.800 | 0.440 | 0.1504 | 21.0220 |

X-ray wavelength used by Scheler formula and Bragg formula: 1.54060 Å

**Table S3.** XRD results of gallbladder neoplasm memristor

| 2θ(°) | Intensity | FWHM | Interplanar spacing (nm) | Scherrer width (nm) |
| --- | --- | --- | --- | --- |
| 37.800 | 11320.511 | 0.580 | 0.2378 | 14.4776 |
| 26.500 | 11150.854 | 0.260 | 0.3361 | 31.3906 |
| 31.720 | 9167.658 | 0.200 | 0.2819 | 41.2934 |
| 51.560 | 6105.805 | 0.320 | 0.1771 | 27.5699 |
| 65.600 | 4424.898 | 0.420 | 0.1422 | 22.5026 |
| 33.740 | 2476.113 | 0.280 | 0.2654 | 29.6484 |
| 44.300 | 1857.173 | 0.360 | 0.2043 | 23.8258 |
| 61.620 | 1786.800 | 0.440 | 0.1504 | 21.0220 |

X-ray wavelength used by Scheler formula and Bragg formula: 1.54060 Å

**Table S4.** Evaluation metrics (Macro-AUC, Accuracy, Kappa, MCC, Macro-F1) for disease classification using memristive electrical parameters.

| Variable | Macro-AUC | Accuracy | Kappa | MCC | Macro_F1 |
| --- | --- | --- | --- | --- | --- |
| Current+(mA) | 0.8333 | 0.6667 | 0.5 | 0.5922 | 0.543 |
| Current-(mA) | 0.8333 | 0.6667 | 0.5 | 0.6031 | 0.5499 |
| Current+/Current- | 0.8333 | 0.6111 | 0.4167 | 0.4304 | 0.592 |
| V_set(V) | 0.8244 | 0.6444 | 0.4667 | 0.5322 | 0.54 |
| V_reset(V) | 0.81 | 0.6889 | 0.5333 | 0.5973 | 0.5957 |
| V_set/V_reset | 0.7267 | 0.5556 | 0.3333 | 0.3861 | 0.4697 |
| HRS | 0.8333 | 0.8 | 0.7 | 0.7047 | 0.798 |
| LRS | 0.8333 | 0.8111 | 0.7167 | 0.72 | 0.8098 |
| HRS/LRS | 0.8333 | 0.9889 | 0.9833 | 0.9835 | 0.9889 |

**Table S5.** Kruskal-Wallis test results for memristor parameters across different groups.

| Variable |  | DF | Prob>Chi-Sq |
| --- | --- | --- | --- |
| Current+(mA) | 79.12414 | 2 | <0.0001 |
| Current-(mA) | 75.20479 | 2 | <0.0001 |
| Current+/Current- | 59.69231 | 2 | <0.0001 |
| V_set(V) | 56.94099 | 2 | <0.0001 |
| V_reset(V) | 63.8778 | 2 | <0.0001 |
| V_set/V_reset | 31.62462 | 2 | <0.0001 |
| HRS | 66.62285 | 2 | <0.0001 |
| LRS | 67.95916 | 2 | <0.0001 |
| HRS/LRS | 79.12348 | 2 | <0.0001 |

**Table S6.** Post-hoc pairwise comparison results for memristor parameters.

| Variable | Comparison pair | Z | Prob | Sig |
| --- | --- | --- | --- | --- |
| Current+(mA) | 0 vs 1 | 4.44757 | <0.0001 | *** |
|  | 0 vs 2 | -4.44757 | <0.0001 | *** |
|  | 1 vs 2 | -8.89514 | <0.0001 | *** |
| Current-(mA) | 0 vs 1 | 3.98298 | 2.0417E-4 | *** |
|  | 0 vs 2 | 8.66273 | <0.0001 | *** |
|  | 1 vs 2 | 4.67975 | <0.0001 | *** |
| Current+/Current- | 0 vs 1 | -0.593 | 1 | - |
|  | 0 vs 2 | -6.96774 | <0.0001 | *** |
|  | 1 vs 2 | -6.37474 | <0.0001 | *** |
| V_set(V) | 0 vs 1 | -0.84509 | 1 | - |
|  | 0 vs 2 | 6.07131 | <0.0001 | *** |
|  | 1 vs 2 | 6.9164 | <0.0001 | *** |
| V_reset(V) | 0 vs 1 | 3.54317 | 0.00119 | ** |
|  | 0 vs 2 | -4.43267 | <0.0001 | *** |
|  | 1 vs 2 | -7.97584 | <0.0001 | *** |
| V_set/V_reset | 0 vs 1 | 5.55937 | <0.0001 | *** |
|  | 0 vs 2 | 2.04585 | 0.12231 | - |
|  | 1 vs 2 | -3.51352 | 0.00133 | ** |
| /HRS | 0 vs 1 | -5.32226 | <0.0001 | *** |
|  | 0 vs 2 | 2.69819 | 0.02092 | * |
|  | 1 vs 2 | 8.02045 | <0.0001 | *** |
| LRS | 0 vs 1 | -2.9354 | 0.00999 | ** |
|  | 0 vs 2 | -8.13905 | <0.0001 | *** |
|  | 1 vs 2 | -5.20366 | <0.0001 | *** |
| HRS/LRS | 0 vs 1 | 4.44755 | <0.0001 | *** |
|  | 0 vs 2 | -4.44755 | <0.0001 | *** |
|  | 1 vs 2 | -8.8951 | <0.0001 | *** |

**Table S7.** Full-cohort Spearman correlations between clinical variables and electrical parameters.

| **Clinical variable** | **I^−^ ρ** | **I^−^ P** | **HRS/LRS ρ** | **HRS/LRS P** |
| --- | --- | --- | --- | --- |
| Age | −0.19 | 0.30 | +0.57 | <0.001 |
| BMI | +0.03 | 0.89 | −0.02 | 0.92 |
| TBil | −0.18 | 0.35 | +0.50 | 0.005 |
| DBil | −0.10 | 0.60 | +0.65 | <0.001 |
| ALT | −0.24 | 0.20 | +0.34 | 0.06 |
| AST | −0.15 | 0.42 | +0.39 | 0.031 |
| ALP | −0.18 | 0.34 | +0.32 | 0.08 |
| GGT | +0.03 | 0.87 | +0.35 | 0.06 |
| WBC | −0.61 | <0.001 | +0.19 | 0.32 |
| CRP | +0.06 | 0.75 | +0.12 | 0.54 |
| CA19-9 | +0.27 | 0.45 | −0.32 | 0.37 |

N = 30 for most variables. BMI and GGT had 29 available values.

**Table S8.** Within-group Spearman ρ values with HRS/LRS for the four variables significant in the full cohort.

| **Variable** | **Full cohort** | **Control** | **Cholelithiasis** | **GBC** |
| --- | --- | --- | --- | --- |
| Age | +0.57 | +0.16 | +0.20 | −0.05 |
| TBil | +0.50 | +0.02 | +0.25 | −0.27 |
| DBil | +0.65 | −0.13 | +0.24 | −0.36 |
| AST | +0.39 | −0.25 | −0.14 | −0.15 |

**Table S9.** Corresponding P values for the within-group correlations in Table S8.

| **Variable** | **Full cohort** | **Control** | **Cholelithiasis** | **GBC** |
| --- | --- | --- | --- | --- |
| Age | <0.001 | 0.65 | 0.58 | 0.89 |
| TBil | 0.005 | 0.96 | 0.49 | 0.45 |
| DBil | <0.001 | 0.73 | 0.50 | 0.31 |
| AST | 0.031 | 0.49 | 0.70 | 0.68 |

**Table S10.** Group means of HRS/LRS and the four clinical variables.

| **Group** | **HRS/LRS** | **Age** | **TBil (μmol/L)** | **DBil (μmol/L)** | **AST (U/L)** |
| --- | --- | --- | --- | --- | --- |
| Control | 4.18 | 57.6 | 43.2 | 24.5 | 58.6 |
| Cholelithiasis | 2.69 | 44.1 | 14.5 | 2.7 | 44.0 |
| GBC | 12.48 | 65.7 | 104.5 | 80.0 | 169.5 |

**Table S11.** Gender distribution by group.

| **Group** | **Male** | **Female** | **Male %** |
| --- | --- | --- | --- |
| Control | 9 | 1 | 90.0 |
| Cholelithiasis | 5 | 5 | 50.0 |
| GBC | 1 | 9 | 10.0 |

**Table S12.** Electrical parameters by sex within the Cholelithiasis group (Mann-Whitney U test).

| **Parameter** | **Male (n = 5) mean ± SD** | **Female (n = 5) mean ± SD** | **MWU P** |
| --- | --- | --- | --- |
| I^−^ (A) | 0.0187 ± 0.0010 | 0.0190 ± 0.0004 | 0.69 |
| HRS/LRS | 2.75 ± 0.16 | 2.63 ± 0.12 | 0.31 |

**Table S13.** Partial Spearman correlations after controlling for sex or age (full cohort, N = 30).

| **Relationship** | **ρ partial** | **P** | **Interpretation** |
| --- | --- | --- | --- |
| Age vs I^−^, sex controlled | −0.27 | 0.16 | No association |
| Sex vs I^−^, age controlled | −0.11 | 0.56 | No association |
| Age vs HRS/LRS, sex controlled | +0.53 | 0.003 | Between-group trend, absent within groups |
| Sex vs HRS/LRS, age controlled | +0.10 | 0.58 | No association |

**Table S14.** Sex-stratified sensitivity analyses: between-group comparisons within each sex.

| **Sex stratum** | **Comparison** | **Parameter** | **Group A**  **(mean ± SD)** | **Group B (mean ± SD)** | **MWU P** | **Cohen's d** |
| --- | --- | --- | --- | --- | --- | --- |
| Male | Control vs Cholelithiasis | I^−^ (A) | 0.0275 ± 0.0031  (n = 9) | 0.0187 ± 0.0010  (n = 5) | 0.001 | -3.36 |
| Male | Control vs Cholelithiasis | HRS/LRS ratio | 4.18 ± 0.39 (n = 9) | 2.75 ± 0.16 (n = 5) | 0.001 | -4.33 |
| Female | Cholelithiasis vs GBC | I^−^ (A) | 0.0190 ± 0.0004  (n = 5) | 0.0064 ± 0.0008  (n = 9) | 0.001 | -19.16 |
| Female | Cholelithiasis vs GBC | HRS/LRS ratio | 2.63 ± 0.12 (n = 5) | 12.41 ± 1.20 (n = 9) | 0.001 | +9.91 |

MWU: two-sided Mann–Whitney U test (exact). d: Cohen's d = (Group B − Group A) / pooled SD. The male stratum reproduces Step 1 of the detection system (control vs pathological); the female stratum reproduces Step 2 (cholelithiasis vs GBC).

**Table S15.** Electrical parameters by age category within each disease group (Mann–Whitney U test).

| **Group** | **Parameter** | **Younger (<60 y) mean ± SD** | **Older (≥60 y) mean ± SD** | **MWU P** |
| --- | --- | --- | --- | --- |
| Control | I⁻ (A) | 0.0267 ± 0.0038 (n = 5) | 0.0283 ± 0.0017 (n = 5) | 0.69 |
|  | HRS/LRS ratio | 4.20 ± 0.49 (n = 5) | 4.15 ± 0.24 (n = 5) | 1.00 |
| Cholelithiasis | I⁻ (A) | 0.0189 ± 0.0008 (n = 9) | 0.0187 (n = 1) | 1.00 |
|  | HRS/LRS ratio | 2.69 ± 0.16 (n = 9) | 2.71 (n = 1) | 0.80 |
| GBC | I⁻ (A) | 0.0061 ± 0.0002 (n = 3) | 0.0065 ± 0.0008 (n = 7) | 0.83 |
|  | HRS/LRS ratio | 12.85 ± 0.40 (n = 3) | 12.32 ± 1.36 (n = 7) | 1.00 |

Within-group comparisons provide the direct test of age effects independent of disease state. MWU denotes the two-sided Mann–Whitney U test (exact), and P = 1.00 arises from ties at small n. The cholelithiasis group contains only one older patient, so that comparison is descriptive. In the full cohort, the age categories differed in the HRS/LRS ratio (4.93 ± 3.85 versus 8.44 ± 4.49, P = 0.009) but not in I⁻ (P = 0.54). This full-cohort pattern mirrors the group gradient, because older patients are predominantly from the GBC group, and it is absent within every group.

**Table S16.** Age-stratified sensitivity analyses. Between-group comparisons within each age stratum.

| **Age stratum** | **Comparison** | **Parameter** | **Group A**  **(mean ± SD)** | **Group B**  **(mean ± SD)** | **MWU P** | **Cohen's d** |
| --- | --- | --- | --- | --- | --- | --- |
| Younger (<60 y) | Control vs Pathological (Step 1) | I⁻ (A) | 0.0267 ± 0.0038 (n = 5) | 0.0157 ± 0.0058  (n = 12) | 0.001 | −2.06 |
|  |  | HRS/LRS ratio | 4.20 ± 0.49  (n = 5) | 5.23 ± 4.60  (n = 12) | 0.13 | +0.26 |
|  | Cholelithiasis vs GBC (Step 2) | I⁻ (A) | 0.0189 ± 0.0008 (n = 9) | 0.0061 ± 0.0002  (n = 3) | 0.009 | −18.26 |
|  |  | HRS/LRS ratio | 2.69 ± 0.16  (n = 9) | 12.85 ± 0.40  (n = 3) | 0.009 | +44.55 |
|  | Control vs Cholelithiasis | I⁻ (A) | 0.0267 ± 0.0038 (n = 5) | 0.0189 ± 0.0008  (n = 9) | 0.001 | −3.41 |
|  |  | HRS/LRS ratio | 4.20 ± 0.49  (n = 5) | 2.69 ± 0.16  (n = 9) | 0.001 | −4.89 |
| Older (≥60 y) | Control vs Pathological (Step 1) | I⁻ (A) | 0.0283 ± 0.0017 (n = 5) | 0.0080 ± 0.0044  (n = 8) | 0.002 | −5.55 |
|  |  | HRS/LRS ratio | 4.15 ± 0.24  (n = 5) | 11.12 ± 3.63  (n = 8) | 0.03 | +2.41 |
|  | Cholelithiasis vs GBC (Step 2) | I⁻ (A) | 0.0187  (n = 1) | 0.0065 ± 0.0008  (n = 7) | 0.25 | n.a. |
|  |  | HRS/LRS ratio | 2.71  (n = 1) | 12.32 ± 1.36  (n = 7) | 0.25 | n.a. |
|  | Control vs Cholelithiasis | I⁻ (A) | 0.0283 ± 0.0017 (n = 5) | 0.0187  (n = 1) | 0.33 | n.a. |
|  |  | HRS/LRS ratio | 4.15 ± 0.24  (n = 5) | 2.71  (n = 1) | 0.33 | n.a. |

Age strata are younger (<60 years) and older (≥60 years), with the cut at 60 years corresponding to the cohort median of 59 years. MWU denotes the two-sided Mann–Whitney U test (exact), with P capped at 0.001 for display. d is Cohen’s d = (Group B − Group A) / pooled SD, not computed for single-patient cells (n.a.). Pathological combines cholelithiasis and GBC patients, corresponding to Step 1 of the detection system, and Step 2 compares cholelithiasis with GBC. The older stratum contains only one cholelithiasis patient, so comparisons involving that group in the older stratum are descriptive.

**Table S17.** Between-group effect sizes of the electrical parameters (Cohen's d with 95% CI).

| **Comparison** | **I^−^ d [95% CI]** | **HRS/LRS d [95% CI]** |
| --- | --- | --- |
| Control vs Cholelithiasis | −4.06 [−5.65, −2.47] | −5.33 [−7.28, −3.38] |
| Cholelithiasis vs GBC | −17.09 [−22.74, −11.44] | +11.86 [+7.89, +15.84] |
| Control vs GBC | −9.89 [−13.24, −6.54] | +9.67 [+6.40, +12.95] |

**Table S18.** Between-group effect sizes of the clinical variables (Cohen's d).

| **Clinical variable** | **Cholelithiasis vs GBC d** | **Control vs GBC d** |
| --- | --- | --- |
| Age | +1.68 | +0.69 |
| TBil | +1.31 | +0.82 |
| DBil | +1.33 | +0.86 |
| WBC | +0.44 | +1.73 |
| CRP | +0.20 | +0.19 |
| ALT | +0.77 | +0.93 |
| AST | +1.17 | +1.05 |
| ALP | +1.13 | +0.54 |

**Table S19.** Individual biliary Cu and Zn concentrations for the thirty patients.

| **Patient** | **Group** | **Biliary Zn (mg/L)** | **Biliary Cu (mg/L)** | **Cu/Zn ratio** |
| --- | --- | --- | --- | --- |
| Control-01 | Control | 4.24 | 3.58 | 0.84 |
| Control-02 | Control | 4.43 | 4.92 | 1.11 |
| Control-03 | Control | 4.62 | 4.05 | 0.88 |
| Control-04 | Control | 6.17 | 4.35 | 0.71 |
| Control-05 | Control | 6.82 | 5.63 | 0.83 |
| Control-06 | Control | 6.54 | 4.85 | 0.74 |
| Control-07 | Control | 3.61 | 3.21 | 0.89 |
| Control-08 | Control | 5.36 | 4.07 | 0.76 |
| Control-09 | Control | 4.81 | 3.88 | 0.81 |
| Control-10 | Control | 8.80 | 6.30 | 0.72 |
| Cholelithiasis-01 | Cholelithiasis | 5.08 | 5.18 | 1.02 |
| Cholelithiasis-02 | Cholelithiasis | 4.79 | 4.54 | 0.95 |
| Cholelithiasis-03 | Cholelithiasis | 5.88 | 3.05 | 0.52 |
| Cholelithiasis-04 | Cholelithiasis | 3.84 | 4.24 | 1.10 |
| Cholelithiasis-05 | Cholelithiasis | 4.65 | 3.59 | 0.77 |
| Cholelithiasis-06 | Cholelithiasis | 8.24 | 8.05 | 0.98 |
| Cholelithiasis-07 | Cholelithiasis | 5.38 | 5.27 | 0.98 |
| Cholelithiasis-08 | Cholelithiasis | 6.28 | 6.11 | 0.97 |
| Cholelithiasis-09 | Cholelithiasis | 4.37 | 4.53 | 1.04 |
| Cholelithiasis-10 | Cholelithiasis | 5.11 | 2.70 | 0.53 |
| GBC-01 | GBC | 3.23 | 5.88 | 1.82 |
| GBC-02 | GBC | 3.36 | 4.10 | 1.22 |
| GBC-03 | GBC | 4.52 | 6.39 | 1.41 |
| GBC-04 | GBC | 2.67 | 7.33 | 2.75 |
| GBC-05 | GBC | 3.69 | 8.51 | 2.31 |
| GBC-06 | GBC | 5.36 | 7.82 | 1.46 |
| GBC-07 | GBC | 3.72 | 6.61 | 1.78 |
| GBC-08 | GBC | 3.08 | 5.01 | 1.63 |
| GBC-09 | GBC | 2.59 | 6.42 | 2.48 |
| GBC-10 | GBC | 2.54 | 7.24 | 2.85 |

Individual concentrations span 2.70 to 8.51 mg/L for Cu and 2.54 to 8.80 mg/L for Zn in bile. Values below the quantification limit would be reported as N.D. (not detected), which did not occur in this cohort.

**Table S20.** Group means of the Cu/Zn variables and the electrical parameters, with between-group Welch t-test P values.

| **Variable** | **Control** | **Cholelithiasis** | **GBC** | **GBC vs Cholelithiasis P** | **GBC vs Control P** |
| --- | --- | --- | --- | --- | --- |
| Biliary Zn (mg/L) | 5.54 | 5.36 | 3.48 | *** | *** |
| Biliary Cu (mg/L) | 4.48 | 4.73 | 6.53 | * | *** |
| Cu/Zn ratio | 0.83 | 0.89 | 1.97 | *** | *** |
| I^−^ (A) | 0.02752 | 0.01885 | 0.00639 | *** | *** |
| HRS/LRS | 4.18 | 2.69 | 12.48 | *** | *** |

Zn, Cu, and Cu/Zn rows come from the Cu/Zn cohort in Table R1-6A. The two electrical rows come from the clinical cohort. I^−^ results are listed first. Significance markers: ***** P < 0.005, **P < 0.01, * P < 0.05**, ns not significant.

**Table S21.** Spearman correlations between the Cu/Zn variables and the two electrical parameters, full cohort and within groups (ρ with P).

| **Variable** | **Outcome** | **Full cohort (n = 30)** | **Control (n = 10)** | **Cholelithiasis (n = 10)** | **GBC (n = 10)** |
| --- | --- | --- | --- | --- | --- |
| Biliary Zn (mg/L) | I^−^ (A) | +0.70 *** | +0.65 * | +0.77 ** | +0.66 * |
| Biliary Zn (mg/L) | HRS/LRS | −0.63 *** | +0.20 ns | −0.66 * | −0.68 * |
| Biliary Cu (mg/L) | I^−^ (A) | −0.53 *** | +0.38 ns | −0.05 ns | −0.37 ns |
| Biliary Cu (mg/L) | HRS/LRS | +0.51 *** | +0.20 ns | +0.12 ns | +0.24 ns |
| Cu/Zn ratio | I^−^ (A) | −0.87 *** | −0.65 * | −0.78 ** | −0.78 ** |
| Cu/Zn ratio | HRS/LRS | +0.68 *** | −0.01 ns | +0.80 ** | +0.84 *** |

I^−^ rows are listed before HRS/LRS rows. Within-group correlations at n = 10 are reported alongside the full-cohort correlations. Significance markers: ***** P < 0.005, ** P < 0.01, * P < 0.05**, ns not significant.

**Table S22**. Monte Carlo power at n = 10 per group (2000 replicates, α = 0.05).

| **Comparison** | **Biliary Zn** | **Biliary Cu** | **Cu/Zn ratio** |
| --- | --- | --- | --- |
| GBC vs Cholelithiasis | 74.7% | 62.0% | 95.3% |
| GBC vs Control | 87.2% | 83.8% | 98.0% |

**Table S23.** Comparison of detection performance for gallbladder lesion discrimination among conventional clinical assays and the proposed memristor platform

| **Method** | **Sensitivity (%)** | **Specificity (%)** | **AUC** | **Accuracy (%)** | **Study Type** | **Sample Size** | **Ref.** |
| --- | --- | --- | --- | --- | --- | --- | --- |
| CA19-9 (>37 U/mL) | 70 | 92 | 0.89 | N/P | Meta-analysis | 4,300 | [1] |
| CA19-9 (GBC vs Benign) | 69 | 91 | N/R | N/P | Meta-analysis subgroup | N/R | [1] |
| Conventional US | 51.6 | 89.7 | 0.706 | 74.5 | Prospective, multi-center | 536 | [2] |
| CEUS | 89.2 | 78.8 | N/R | 84.9 | Retrospective | 151 | [3] |
| CECT | 85.7 | 74.3 | N/R | 80.5 | Retrospective | 151 | [3] |
| MRI (DWI) | 64 | 75 | N/R | N/P | Meta-analysis | 592 | [4] |
| MRCP | 92.5 | 80 | N/R | 91.1 | Prospective | 45 | [5] |
| Memristor 2-Step (This Study) | 100 | 95 | 1 | 96.7 | Patient-level LOOCV | 30 | This study |

N/P: not pooled (prevalence-dependent). N/R: not reported.

LOOCV: leave-one-out cross-validation;

GBC: gallbladder carcinoma;

US: ultrasound;

CEUS: contrast-enhanced ultrasound;

CECT: contrast-enhanced computed tomography;

MRI (DWI): diffusion-weighted magnetic resonance imaging;

MRCP: magnetic resonance cholangiopancreatography.

**Table S24.** Comparison of the laboratory device tested in this study and the conceptual future cartridge.

| **Feature** | **Laboratory device (tested in this study)** | **Conceptual future cartridge** |
| --- | --- | --- |
| Active switching layer | Patient bile, spin-coated onto FTO | Patient bile, capillary-filled at the point of care |
| Bile deposition | Two-step spin coating (500 rpm, 10 s; 2,500 rpm, 30 s) | Injection of ~50–100 μL into an empty chamber of a few microliters |
| Thermal treatment | Annealing at 30 °C for 10 min | None (room temperature) |
| Electrodes | Ag deposited by magnetron sputtering after film formation | Ag and FTO pre-patterned on the chamber walls during manufacture; bile fills the micrometer-scale gap |
| Fluidics | Open planar substrate | Sealed chip with inlet port (Luer-style connection to the ERCP catheter) and vent outlet; capillary filling, no pump |
| Measurement | Semiconductor parameter analyzer, 0 V → +2.0 V → −2.0 V → 0 V sweep, 0.05 V steps, room temperature | Portable reader applying the same sweep protocol (conceptual) |
| Sample handling | 2–5 mL bile collected; film formed in the laboratory | ~50–100 μL injected per test; no laboratory steps at the bedside |
| Status | Physically tested (30 patients, 90 devices) | Not built or tested; conceptual design requiring engineering development |

**Table S25.** De-identified patient-level dataset for the pilot cohort (N = 30: 10 control, 10 cholelithiasis, and 10 GBC patients). Panel a: clinical variables, bile chemistry, and tumor stage. Panel b: patient-level electrical features and leave-one-patient-out cross-validation (LPOCV) predictions.

**Table S25 (panel a)**

| **Patient code** | **Group** | **Age (y)** | **Gender** | **BMI (kg/m²)** | **ALT (U/L)** | **AST (U/L)** | **ALP (U/L)** | **GGT (U/L)** | **TBil (μmol/L)** | **DBil (μmol/L)** | **WBC (×10⁹/L)** | **CRP (mg/L)** | **CA19-9 (U/mL)** | **Tumor stage/**  **disease label** | **Cu (mg/L)** | **Zn (mg/L)** | **Cu/Zn** |
| --- | --- | --- | --- | --- | --- | --- | --- | --- | --- | --- | --- | --- | --- | --- | --- | --- | --- |
| Control-01 | Control | 45 | M | 17.9931 | 34 | 40 | 105 | 75 | 13.2 | 6.3 | 3.53 | 7.9 | <2 | Hepatic Hemangioma | 3.58 | 4.24 | 0.84 |
| Control-02 | Control | 75 | M | 22.49 | 104 | 96 | 194 | 346 | 12.1 | 4.4 | 5.19 | 36.7 | <2 | Hepatic Hemangioma | 4.92 | 4.43 | 1.11 |
| Control-03 | Control | 63 | M |  | 22 | 21 | 120 | 102 | 8.8 | 4.7 | 4.41 | 24.6 | 10.4 | Hepatic Hemangioma | 4.05 | 4.62 | 0.88 |
| Control-04 | Control | 60 | M | 24.9135 | 40 | 51 | 134 | 168 | 39 | 9.1 | 4.88 | 17.1 | 35.9 | Hepatic Hemangioma | 4.35 | 6.17 | 0.71 |
| Control-05 | Control | 71 | M | 26.5731 | 81 | 78 | 95 | 52 | 27.8 | 7 | 2.75 | 1.7 | <2 | Hepatic Hemangioma | 5.63 | 6.82 | 0.83 |
| Control-06 | Control | 48 | M | 18.6122 | 21 | 55 | 660 | 777 | 139 | 121.8 | 5.74 | 25.8 | <2 | Hepatic Hemangioma | 4.85 | 6.54 | 0.74 |
| Control-07 | Control | 34 | M | 27.0416 | 49 | 36 | 77 | 38 | 29.6 | 4.9 | 5.15 | 3.8 | <2 | Hepatic Hemangioma | 3.21 | 3.61 | 0.89 |
| Control-08 | Control | 70 | M | 18.3594 | 39 | 39 | 98 | 91 | 16.3 | 7.6 | 4.22 | 4.3 | <2 | Hepatic Hemangioma | 4.07 | 5.36 | 0.76 |
| Control-09 | Control | 59 | F | 22.2656 | 227 | 133 | 423 | 495 | 106.1 | 69.9 | 4.24 | 5.1 | <2 | Hepatic Hemangioma | 3.88 | 4.81 | 0.81 |
| Control-10 | Control | 51 | M | 23.4375 | 31 | 37 | 61 | 46 | 39.6 | 9 | 3.21 | 2.6 | <2 | Hepatic Hemangioma | 6.3 | 8.8 | 0.72 |
| Cholelithiasis-01 | Cholelithiasis | 36 | F | 21.2306 | 14 | 23 | 32 | 17 | 18.1 | 0.1 | 3.15 | 4.2 | <2 | Cholelithiasis | 5.18 | 5.08 | 1.02 |
| Cholelithiasis-02 | Cholelithiasis | 73 | F | 20.44 | 12 | 18 | 82 | 10 | 11.8 | 2.8 | 4.84 | 2.3 | <2 | Cholelithiasis | 4.54 | 4.79 | 0.95 |
| Cholelithiasis-03 | Cholelithiasis | 27 | M | 23.8751 | 51 | 34 | 99 | 38 | 16.4 | 3.5 | 4.44 | 2.9 | <2 | Cholelithiasis | 3.05 | 5.88 | 0.52 |
| Cholelithiasis-04 | Cholelithiasis | 34 | M | 29.827 | 57 | 27 | 68 | 65 | 15.2 | 4.4 | 4.88 | 1.8 | <2 | Cholelithiasis | 4.24 | 3.84 | 1.1 |
| Cholelithiasis-05 | Cholelithiasis | 34 | F | 21.1938 | 14 | 19 | 39 | 11 | 20.9 | 2.2 | 13.63 | 4.8 | 5.4 | Cholelithiasis | 3.59 | 4.65 | 0.77 |
| Cholelithiasis-06 | Cholelithiasis | 55 | M | 25.5102 | 20 | 17 | 117 | 29 | 12 | 1.6 | 9.3 | 9 | <2 | Cholelithiasis | 8.05 | 8.24 | 0.98 |
| Cholelithiasis-07 | Cholelithiasis | 36 | M | 21.7993 | 312 | 109 | 153 | 197 | 15.7 | 3.9 | 7.59 | 84.7 | <2 | Cholelithiasis | 5.27 | 5.38 | 0.98 |
| Cholelithiasis-08 | Cholelithiasis | 58 | F | 25.9645 | 263 | 148 | 146 | 125 | 13.2 | 4.1 | 6.34 | 3.1 | <2 | Cholelithiasis | 6.11 | 6.28 | 0.97 |
| Cholelithiasis-09 | Cholelithiasis | 55 | M | 20.7612 | 40 | 28 | 60 | 14 | 17.6 | 4.4 | 4.5 | 3.7 | 6.3 | Cholelithiasis | 4.53 | 4.37 | 1.04 |
| Cholelithiasis-10 | Cholelithiasis | 33 | F | 21.9735 | 11 | 17 | 80 | 10 | 4.3 | 0.4 | 5.51 | 1.6 | <2 | Cholelithiasis | 2.7 | 5.11 | 0.53 |
| GBC-01 | GBC | 77 | F | 20.3954 | 548 | 336 | 918 | 436 | 193 | 165.4 | 7.15 | 24.5 | 8490 | T3N1M0 | 5.88 | 3.23 | 1.82 |
| GBC-02 | GBC | 69 | F | 26.6728 | 122 | 331 | 125 | 86 | 27.4 | 12.1 | 8.66 | 4.9 | 89.8 | T3N2M0 | 4.1 | 3.36 | 1.22 |
| GBC-03 | GBC | 68 | F | 22.0158 | 337 | 220 | 495 | 511 | 209.9 | 182.2 | 5.77 | 12.3 | 9005 | T2N0M0 | 6.39 | 4.52 | 1.41 |
| GBC-04 | GBC | 43 | F | 26.8923 | 25 | 20 | 81 | 96 | 13.2 | 3.6 | 5.82 | 4.1 | 9.1 | pT1BN0M0 | 7.33 | 2.67 | 2.75 |
| GBC-05 | GBC | 59 | F | 27.4348 | 75 | 74 | 177 | 48 | 229 | 164.9 | 5.19 | 2.8 | 1918 | T3N1M0 | 8.51 | 3.69 | 2.31 |
| GBC-06 | GBC | 59 | F | 26.0146 | 13 | 22 | 119 | 12 | 13.5 | 7 | 4.26 | 1.7 | 61.4 | pT3N1M0 | 7.82 | 5.36 | 1.46 |
| GBC-07 | GBC | 76 | M | 19.8177 | 283 | 273 | 722 | 582 | 218.9 | 174.9 | 12.9 | 100.5 | 15.7 | T2N0M0 | 6.61 | 3.72 | 1.78 |
| GBC-08 | GBC | 76 | F | 18.3594 | 18 | 28 | 54 | 32 | 17.8 | 4.1 | 8.68 | 2.3 | 12.2 | T3N1M0 | 5.01 | 3.08 | 1.63 |
| GBC-09 | GBC | 64 | F | 20.7008 | 588 | 349 | 534 |  | 98.9 | 79 | 8.87 | 3.2 | 96.8 | T3N2M0 | 6.42 | 2.59 | 2.48 |
| GBC-10 | GBC | 66 | F | 21.6889 | 120 | 42 | 140 | 135 | 23 | 7 | 9.43 | 16.6 | 219 | pT3N0M0 | 7.24 | 2.54 | 2.85 |

**Table S25 (panel b)**

| **Patient code** | **Group** | **I⁻ mean (A)** | **I⁻ SD (A)** | **HRS/LRS mean** | **HRS/LRS SD** | **S1 cutoff (A)** | **S1 pred** | **S2 cutoff** | **S2 pred** | **Final pred** | **Correct** |
| --- | --- | --- | --- | --- | --- | --- | --- | --- | --- | --- | --- |
| Control-01 | Control | 0.0204 | 0.0024 | 3.8542 | 0.3671 | 0.0259 | Disease |  |  | Cholelithiasis | MISS |
| Control-02 | Control | 0.0259 | 0.0005 | 4.1698 | 0.089 | 0.0204 | Control |  |  | Control | OK |
| Control-03 | Control | 0.0272 | 0.0005 | 3.9425 | 0.1152 | 0.0204 | Control |  |  | Control | OK |
| Control-04 | Control | 0.0296 | 0.0022 | 3.9273 | 0.1315 | 0.0204 | Control |  |  | Control | OK |
| Control-05 | Control | 0.0288 | 0.0045 | 4.192 | 0.1128 | 0.0204 | Control |  |  | Control | OK |
| Control-06 | Control | 0.0266 | 0.006 | 3.7049 | 0.3254 | 0.0204 | Control |  |  | Control | OK |
| Control-07 | Control | 0.0279 | 0.0003 | 4.3241 | 0.1022 | 0.0204 | Control |  |  | Control | OK |
| Control-08 | Control | 0.0302 | 0.0017 | 4.5297 | 0.7427 | 0.0204 | Control |  |  | Control | OK |
| Control-09 | Control | 0.0279 | 0.0014 | 4.1816 | 0.0247 | 0.0204 | Control |  |  | Control | OK |
| Control-10 | Control | 0.0306 | 0.0102 | 4.9574 | 0.6694 | 0.0204 | Control |  |  | Control | OK |
| Cholelithiasis-01 | Cholelithiasis | 0.0187 | 0.0005 | 2.7964 | 0.0699 | 0.0204 | Disease | 2.967 | Cholelithiasis | Cholelithiasis | OK |
| Cholelithiasis-02 | Cholelithiasis | 0.0187 | 0.0001 | 2.7115 | 0.0304 | 0.0204 | Disease | 2.967 | Cholelithiasis | Cholelithiasis | OK |
| Cholelithiasis-03 | Cholelithiasis | 0.0199 | 0.0002 | 2.5971 | 0.0402 | 0.0204 | Disease | 2.967 | Cholelithiasis | Cholelithiasis | OK |
| Cholelithiasis-04 | Cholelithiasis | 0.0173 | 0.0006 | 2.967 | 0.1093 | 0.0204 | Disease | 2.8744 | GBC | GBC | MISS |
| Cholelithiasis-05 | Cholelithiasis | 0.0187 | 0.0002 | 2.6298 | 0.0244 | 0.0204 | Disease | 2.967 | Cholelithiasis | Cholelithiasis | OK |
| Cholelithiasis-06 | Cholelithiasis | 0.0194 | 0.0001 | 2.6642 | 0.0226 | 0.0204 | Disease | 2.967 | Cholelithiasis | Cholelithiasis | OK |
| Cholelithiasis-07 | Cholelithiasis | 0.0186 | 0.0002 | 2.6523 | 0.0309 | 0.0204 | Disease | 2.967 | Cholelithiasis | Cholelithiasis | OK |
| Cholelithiasis-08 | Cholelithiasis | 0.0194 | 0.0003 | 2.493 | 0.0074 | 0.0204 | Disease | 2.967 | Cholelithiasis | Cholelithiasis | OK |
| Cholelithiasis-09 | Cholelithiasis | 0.0183 | 0.0012 | 2.8744 | 0.2396 | 0.0204 | Disease | 2.967 | Cholelithiasis | Cholelithiasis | OK |
| Cholelithiasis-10 | Cholelithiasis | 0.0194 | 0.0004 | 2.5398 | 0.0397 | 0.0204 | Disease | 2.967 | Cholelithiasis | Cholelithiasis | OK |
| GBC-01 | GBC | 0.0061 | 0 | 13.181 | 0.1284 | 0.0204 | Disease | 2.967 | GBC | GBC | OK |
| GBC-02 | GBC | 0.0076 | 0.0003 | 10.5435 | 0.5508 | 0.0204 | Disease | 2.967 | GBC | GBC | OK |
| GBC-03 | GBC | 0.0079 | 0.0014 | 10.1329 | 2.3577 | 0.0204 | Disease | 2.967 | GBC | GBC | OK |
| GBC-04 | GBC | 0.006 | 0.0001 | 13.2896 | 0.1353 | 0.0204 | Disease | 2.967 | GBC | GBC | OK |
| GBC-05 | GBC | 0.006 | 0 | 12.7436 | 0.1593 | 0.0204 | Disease | 2.967 | GBC | GBC | OK |
| GBC-06 | GBC | 0.0063 | 0.0002 | 12.5074 | 0.4329 | 0.0204 | Disease | 2.967 | GBC | GBC | OK |
| GBC-07 | GBC | 0.0061 | 0.0002 | 13.1117 | 0.2769 | 0.0204 | Disease | 2.967 | GBC | GBC | OK |
| GBC-08 | GBC | 0.006 | 0 | 13.0282 | 0.1131 | 0.0204 | Disease | 2.967 | GBC | GBC | OK |
| GBC-09 | GBC | 0.0059 | 0.0002 | 13.0421 | 0.2092 | 0.0204 | Disease | 2.967 | GBC | GBC | OK |
| GBC-10 | GBC | 0.006 | 0.0001 | 13.2318 | 0.0755 | 0.0204 | Disease | 2.967 | GBC | GBC | OK |

I⁻ mean and I⁻ SD are the patient-level arithmetic mean and standard deviation of three technical-replicate device measurements of the negative peak current. HRS/LRS mean and SD are the corresponding values for the HRS/LRS ratio. LPOCV, leave-one-patient-out cross-validation. S1 cutoff and S2 cutoff are the Youden's J optimal cutoffs derived within the training fold of the respective patient, i.e., with that patient excluded from training. S1 pred, S2 pred, and Final pred are the corresponding out-of-fold predictions, and Correct indicates agreement with the true label. Blank cells denote values not recorded in the study database (The excessively low CA19-9 value results in only displaying range values (<2). Tumor stage applies to GBC patients only). The liver function values were measured on the first day after surgery.

**Table S26.** Demographic, clinical, bile chemistry, and electrical characteristics of the pilot cohort by group.

| **Variable** | **Control (n = 10)** | **Cholelithiasis (n = 10)** | **GBC (n = 10)** | **P** |
| --- | --- | --- | --- | --- |
| Age (y) | 57.6 ± 13.0 | 44.1 ± 15.0 | 65.7 ± 10.3 | 0.0065 |
| Gender, male n (%) | 9 (90) | 5 (50) | 1 (10) | 0.0016 |
| BMI (kg/m²) | 22.4 ± 3.5 (n = 9) | 23.3 ± 3.0 | 23.0 ± 3.4 | 0.96 |
| ALT (U/L) | 65 ± 63 | 79 ± 112 | 213 ± 217 | 0.15 |
| AST (U/L) | 59 ± 34 | 44 ± 46 | 170 ± 145 | 0.03 |
| ALP (U/L) | 197 ± 193 | 88 ± 41 | 336 ± 308 | 0.04 |
| GGT (U/L) | 219 ± 247 | 52 ± 62 | 215 ± 227 (n = 9) | 0.02 |
| TBil (μmol/L) | 43.2 ± 43.9 | 14.5 ± 4.6 | 104.5 ± 96.8 | 0.02 |
| DBil (μmol/L) | 24.5 ± 39.6 | 2.7 ± 1.6 | 80.0 ± 82.2 | 0.001 |
| WBC (×10⁹/L) | 4.33 ± 0.95 | 6.42 ± 3.08 | 7.67 ± 2.56 | 0.0046 |
| CRP (mg/L) | 13.0 ± 12.3 | 11.8 ± 25.7 | 17.3 ± 30.2 | 0.32 |
| CA19-9 (U/mL), median (IQR) | N/R | N/R | 93.3 (27.1–1493.3) (n = 10) | n.a. |
| Cu (mg/L) | 4.48 ± 0.95 | 4.73 ± 1.56 | 6.53 ± 1.31 | 0.0051 |
| Zn (mg/L) | 5.54 ± 1.55 | 5.36 ± 1.23 | 3.48 ± 0.90 | 0.0016 |
| Cu/Zn ratio | 0.83 ± 0.12 | 0.89 ± 0.21 | 1.97 ± 0.58 | 0.001 |
| I⁻ (A) | 0.0275 ± 0.0029 | 0.0188 ± 0.0007 | 0.0064 ± 0.0007 | 0.001 |
| HRS/LRS ratio | 4.18 ± 0.37 | 2.69 ± 0.15 | 12.48 ± 1.16 | 0.001 |

Values are mean ± SD unless stated otherwise. P values are from the Kruskal–Wallis test across the three groups for continuous variables and from the Fisher exact test for gender. CA19-9 was recorded for all ten GBC patients and is given as median (IQR). In the control and cholelithiasis groups, most values were below the lower limit of quantification (<2 U/mL) with only sporadic measurable values (6.3–35.9 U/mL); summary statistics and between-group comparisons were therefore not performed for these groups. BMI was missing for one control patient and GGT for one GBC patient (n annotated). I⁻, negative peak current; HRS/LRS, high- to low-resistance state ratio; GBC, gallbladder carcinoma.

**Table S27.** Bile Compositional Differences Across Three Pathophysiological States

| **Molecular Category** | **Specific Constituent** | **No Disease State** | **Cholelithiasis State** | **Gallbladder Carcinoma State** | **Reference** |
| --- | --- | --- | --- | --- | --- |
| Electrolytes | Na⁺ | 141–165 mmol/L | High interindividual variability, related to gallbladder concentrating function | Limited individual quantification; total electrolytes decreased | [6, 7] |
|  | Cl⁻ | 77–117 mmol/L | Possibly elevated (animal models suggest impaired NaCl reabsorption; direct human data limited) | Limited data | [8, 9] |
|  | Ca^2+^ | 1.25–3.2 mmol/L | — | — | [6] |
|  | HCO₃⁻ | 12–55 mmol/L | — | — | [6] |
|  | pH | ～6.9–7.0 | Elevated (~7.09, alkaline) | Limited data | [10] |
| Trace Elements | Cu | Relatively low | Moderately elevated | Significantly elevated | [11-13] |
|  | Zn | Relatively high | Relatively low | Significantly decreased | [11, 12] |
|  | Cu/Zn Ratio | ~1.11 (serum) | ~1.35 (serum) | ~2.12 (serum) | [11] |
| Organic Constituents - Bile Acids（BA） | Total Bile Acids | Control | Relatively high (preserved concentrating function) | Significantly decreased | [14] |
|  | Secondary Bile Acids (DCA/LCA) | Control proportion | Possibly elevated proportion (inferred from enterohepatic circulation; direct human bile data limited) | Decreased proportion | [14-16] |
|  | Primary/Secondary Bile Acid Ratio | Control | Possibly decreased | Elevated | [14] |
| Organic Constituents - Lipids | Phospholipids (mainly PC) | 25–810 mg/100 mL | Relatively deficient | Significantly decreased (lipidomics: 384 down-regulated among 466 DELs) | [14, 17] |
|  | Cholesterol | 60–320 mg/100 mL | Supersaturated (CSI significantly elevated) | Decreased total lipid concentration | [16, 17] |
|  | Phospholipid/BA Ratio | ~0.3 | Decreased | Decreased | [6] |
|  | Cholesterol/BA Ratio | ~0.07 | Elevated | — | [17] |
|  | 4-HNE (lipid peroxidation product) | Low | — | Elevated | [14] |
| Organic Constituents - Proteins | Total Protein | 1–3 mg/mL | — | — | [18] |
|  | Mucin | Low | Hypersecreted | — | [16] |

**Text S1**

1. The direct tunneling and Fowler–Nordheim tunneling are derived from the Simmons equation and correspond to the following Equations (1) and (2), respectively.[19]

$I \propto V\cdot e^{\frac{-2d\cdot\sqrt{2m^{*}\Phi_{B}}}{h}}$	(1)

$I \propto V^{2}\cdot e^{\frac{-4d\cdot\sqrt{2m^{*}\Phi_{B}^{3}}}{3hqv}}$	(2)

where *d* represents the barrier width, *m^*^* represents the electron effective mass, ***Ф_B_*** represents the barrier height, *q* represents the electron charge, and *h* is represents Planck’s constant.

2. The Schottky emission can be expressed as the following equation. [20]

$J_{se}= \frac{4\pi qm^{*}{(kT)}^{2}}{h^{3}}\cdot e^{\frac{-q(\varphi-\sqrt{\frac{qV}{4\pi\varepsilon}})}{kT}}$	(3)

where *q* and *m** represent the elementary charge and effective mass of the electron. *k* and *T* are the elementary charge and the absolute temperature, respectively. *ε* and *φ* represent the permittivity and junction barrier height, respectively. *V* is the electric field, and *h* is Planck’s constant.

3. The hopping conduction can be expressed as the following equation. [21]

$J_{hopping}=V\cdot e^{\frac{-E_{a}}{kT}}$	(4)

where *E_a_* is the electron concentration in the conduction band of the functional layer.

4. The Ohmic conduction model can be described by the following equation: [22]

$J_{ohmic}=q\mu N_{c}V\exp\left[\frac{-(E_{c}-E_{F})}{kT}\right]$ (5)

where *q* is the elementary charge, *μ* denotes the mobility of the charge carriers, *N_c_* is the effective density of states in the conduction band, *V* is the applied voltage or electric field. The *E_c_* and *E_F_* represent the conduction band edge and the Fermi energy level, respectively. *k* is the Boltzmann constant, and *T* is the absolute temperature.

5. The Poole-Frenkel (PF) emission can be described by the following equation: [23]

 (6)

where *N*_c_ is the density of states.

6. Scherrer width calculation formula can be expressed as the following equation:

$D=\frac{K\cdot\lambda}{\beta\cdot\cos\theta}$	(5)

The crystallite size (D, unit: nm) is calculated by Scheler formula. The shape factor (k) is 0.90. The X-ray wavelength (λ, unit: nm) is the commonly used Cu-Kα ray, and its wavelength is fixed at 0.154060 nm. The diffraction peak half-maximum width (β) required in the formula needs to be substituted in radians, and the diffraction angle (θ) also needs to be converted into radians to participate in the calculation.

**Reference**

1. X. Zhou, "Meta-analysis of the diagnostic performance of serum carbohydrate antigen 19-9 for the detection of gallbladder cancer," Int J Biol Markers 37, no. 1 (2022): 81. <https://doi.org/10.1177/17246008211068866>

2. L. F. Wang, Q. Wang, F. Mao, et al., "Risk stratification of gallbladder masses by machine learning-based ultrasound radiomics models: a prospective and multi-institutional study," Eur Radiol 33, no. 12 (2023): 8899. <https://doi.org/10.1007/s00330-023-09891-8>

3. L. Fang, Z. Qiu, L. Xu, et al., "Comparative study of contrast-enhanced ultrasound and contrast-enhanced computed tomography in the diagnosis of benign and malignant lesions of the gallbladder," Drug Discov Ther 19, no. 6 (2026): 414. <https://doi.org/10.5582/ddt.2025.01105>

4. H. Kuipers, F. J. Hoogwater, G. A. Holtman, et al., "Clinical value of diffusion-weighted MRI for differentiation between benign and malignant gallbladder disease: a systematic review and meta-analysis," Acta Radiol 62, no. 8 (2021): 987. <https://doi.org/10.1177/0284185120950115>

5. S. Sharma, Y. Pimpalwar, S. Kumar Srivastava, et al., "Correlative Evaluation of Transabdominal Ultrasonography and Magnetic Resonance Cholangiopancreatography Findings in Patients with Periampullary and Biliary System Pathologies," Journal of Neonatal Surgery 14, no. 30S (2025): 1062. <https://www.jneonatalsurg.com/index.php/jns/article/view/8470>

6. J. L. Boyer, "Bile formation and secretion," Compr Physiol 3, no. 3 (2013): 1035. <https://doi.org/10.1002/cphy.c120027>

7. A. Esteller, "Physiology of bile secretion," World J Gastroenterol 14, no. 37 (2008): 5641. <https://doi.org/10.3748/wjg.14.5641>

8. M. Dijkstra, F. Kuipers, E. P. Smit, et al., "Biliary secretion of trace elements and minerals in the rat. Effects of bile flow variation and diurnal rhythms," J Hepatol 13, no. 1 (1991): 112. <https://doi.org/10.1016/0168-8278(91)90872-9>

9. K. Matsumoto, M. Imasato, Y. Yamazaki, et al., "Claudin 2 deficiency reduces bile flow and increases susceptibility to cholesterol gallstone disease in mice," Gastroenterology 147, no. 5 (2014): 1134. <https://doi.org/10.1053/j.gastro.2014.07.033>

10. G. R. Verma, A. K. Pandey, S. M. Bose, et al., "Study of serum calcium and trace elements in chronic cholelithiasis," ANZ J Surg 72, no. 8 (2002): 596. <https://doi.org/10.1046/j.1445-2197.2002.02485.x>

11. S. K. Gupta, S. P. Singh, V. K. Shukla, "Copper, zinc, and Cu/Zn ratio in carcinoma of the gallbladder," J Surg Oncol 91, no. 3 (2005): 204. <https://doi.org/10.1002/jso.20306>

12. N. Sharma, M. Yadav, G. Tripathi, et al., "Bile multi-omics analysis classifies lipid species and microbial peptides predictive of carcinoma of gallbladder," Hepatology 76, no. 4 (2022): 920. <https://doi.org/10.1002/hep.32496>

13. G. W. Evans, "Copper homeostasis in the mammalian system," Physiol Rev 53, no. 3 (1973): 535. <https://doi.org/10.1152/physrev.1973.53.3.535>

14. Y. C. Li, K. S. Li, Z. L. Liu, et al., "Research progress of bile biomarkers and their immunoregulatory role in biliary tract cancers," Front Immunol 13, (2022): 1049812. <https://doi.org/10.3389/fimmu.2022.1049812>

15. P. Portincasa, A. Moschetta, G. Palasciano, "Cholesterol gallstone disease," Lancet 368, no. 9531 (2006): 230. <https://doi.org/10.1016/s0140-6736(06)69044-2>

16. K. J. Maurer, M. C. Carey, J. G. Fox, "Roles of infection, inflammation, and the immune system in cholesterol gallstone formation," Gastroenterology 136, no. 2 (2009): 425. <https://doi.org/10.1053/j.gastro.2008.12.031>

17. D. Q. Wang, D. E. Cohen, M. C. Carey, "Biliary lipids and cholesterol gallstone disease," J Lipid Res 50 Suppl, no. Suppl (2009): S406. <https://doi.org/10.1194/jlr.R800075-JLR200>

18. P. R. Harvey, G. A. Upadhya, J. L. Toth, et al., "Fluorometric assay of protein in native human bile," Clin Chim Acta 183, no. 2 (1989): 147. <https://doi.org/10.1016/0009-8981(89)90330-6>

19. J. H. Sung, J. H. Park, D. S. Jeon, et al., "Retention enhancement through capacitance-dependent voltage division analysis in 3D stackable TaOx/HfO2-based selectorless memristor," Materials & Design 207, (2021): 109845. <https://doi.org/https://doi.org/10.1016/j.matdes.2021.109845>

20. S. Choi, Y. Kim, T. Van Nguyen, et al., "Low-Power Self-Rectifying Memristive Artificial Neural Network for Near Internet-of-Things Sensor Computing," Advanced Electronic Materials 7, no. 6 (2021): 2100050. <https://doi.org/https://doi.org/10.1002/aelm.202100050>

21. E. Punkka, M. F. Rubner, J. D. Hettinger, et al., "TUNNELING AND HOPPING CONDUCTION IN LANGMUIR-BLODGETT THIN-FILMS OF POLY(3-HEXYLTHIOPHENE)," PHYSICAL REVIEW B 43, no. 11 (1991): 9076. <https://doi.org/10.1103/PhysRevB.43.9076>

22. F. C. Chiu, P. W. Li, W. Y. Chang, "Reliability characteristics and conduction mechanisms in resistive switching memory devices using ZnO thin films," Nanoscale Research Letters 7, no. 1 (2012): 178.

23. E. W. Lim, R. Ismail, "Conduction Mechanism of Valence Change Resistive Switching Memory: A Survey," Electronics 4, (2015): 586. <https://doi.org/10.3390/electronics4030586>
